# Compensatory cross-talk between autophagy and glycolysis regulates senescence and stemness in heterogeneous glioblastoma tumor subpopulations

**DOI:** 10.1186/s40478-023-01604-y

**Published:** 2023-07-07

**Authors:** Emma Martell, Helgi Kuzmychova, Harshal Senthil, Esha Kaul, Chirayu R. Chokshi, Chitra Venugopal, Christopher M. Anderson, Sheila K. Singh, Tanveer Sharif

**Affiliations:** 1 Department of Pathology, Rady Faculty of Health Sciences, University of Manitoba, Winnipeg, MB Canada; 2 Department of Human Anatomy and Cell Science, Rady Faculty of Health Sciences, University of Manitoba, Winnipeg, MB Canada; 3 Faculty of Science, University of Manitoba, Winnipeg, MB Canada; 4 Department of Biochemistry, McMaster University, Hamilton, ON Canada; 5 Department of Surgery, McMaster University, Hamilton, ON Canada; 6 Department of Pharmacology and Therapeutics, Rady Faculty of Health Sciences, University of Manitoba, Winnipeg, MB Canada; 7 Neuroscience Research Program, Kleysen Institute for Advanced Medicine, Health Sciences Centre, Winnipeg, MB Canada

**Keywords:** Glioblastoma, Tumor heterogeneity, Cancer stem cell-like cells, Metabolism, Glycolysis, Autophagy, Senescence

## Abstract

Despite tremendous research efforts, successful targeting of aberrant tumor metabolism in clinical practice has remained elusive. Tumor heterogeneity and plasticity may play a role in the clinical failure of metabolism-targeting interventions for treating cancer patients. Moreover, compensatory growth-related processes and adaptive responses exhibited by heterogeneous tumor subpopulations to metabolic inhibitors are poorly understood. Here, by using clinically-relevant patient-derived glioblastoma (GBM) cell models, we explore the cross-talk between glycolysis, autophagy, and senescence in maintaining tumor stemness. We found that stem cell-like GBM tumor subpopulations possessed higher basal levels of glycolytic activity and increased expression of several glycolysis-related enzymes including, GLUT1/SLC2A1, PFKP, ALDOA, GAPDH, ENO1, PKM2, and LDH, compared to their non-stem-like counterparts. Importantly, bioinformatics analysis also revealed that the mRNA expression of glycolytic enzymes positively correlates with stemness markers (*CD133/PROM1* and *SOX2*) in patient GBM tumors. While treatment with glycolysis inhibitors induced senescence in stem cell-like GBM tumor subpopulations, as evidenced by increased β-galactosidase staining and upregulation of the cell cycle regulators p21^Waf1/Cip1^/*CDKN1A* and p16^INK4A^/*CDKN2A*, these cells maintained their aggressive stemness features and failed to undergo apoptotic cell death. Using various techniques including autophagy flux and EGFP-MAP1LC3B^+^ puncta formation analysis, we determined that inhibition of glycolysis led to the induction of autophagy in stem cell-like GBM tumor subpopulations, but not in their non-stem-like counterparts. Similarly, blocking autophagy in stem cell-like GBM tumor subpopulations induced senescence-associated growth arrest without hampering stemness capacity or inducing apoptosis while reciprocally upregulating glycolytic activity. Combinatorial treatment of stem cell-like GBM tumor subpopulations with autophagy and glycolysis inhibitors blocked the induction of senescence while drastically impairing their stemness capacity which drove cells towards apoptotic cell death. These findings identify a novel and complex compensatory interplay between glycolysis, autophagy, and senescence that helps maintain stemness in heterogeneous GBM tumor subpopulations and provides a survival advantage during metabolic stress.

**Supplementary Information:**

The online version contains supplementary material available at 10.1186/s40478-023-01604-y.

## Introduction

Tumors exist as heterogeneous entities that contain diverse subpopulations of cells at varying stages of differentiation and proliferative capacity and these functional differences are driven by complex genomic, epigenomic, and transcriptomic intra-tumoral heterogeneity [28, 34, 38, 41]. Glioblastoma (GBM) is the most aggressive primary malignant brain tumor in adults, characterized by a 5-year survival rate of approximately 5% due to inevitable disease relapse [23, 55, 56]. GBM tumors exhibit immense heterogeneity and contain ‘stem cell-like’ tumor subpopulations that are resistant to conventional therapies and have aggressive tumor seeding capacity, which contributes to the poor prognosis of this disease [2, 21, 40, 41].

Treating cancer by targeting their abnormal metabolic phenotypes has been a widely sought-after concept for many years [15, 42, 44, 66]. Yet, poor efficacy and dose-limiting toxicities demonstrated by metabolism-targeting agents in clinical trials has prevented wide-spread use of metabolic inhibitors in standard of care treatment regimens for cancer patients [11, 29, 53, 69]. GBM tumors tend to exhibit a high dependency on the ‘Warburg effect’, a metabolic phenotype that describes the enhanced rate of fermentative glycolysis utilized by cancer cells as compared to normal tissues [68]. Despite this, clinical trials utilizing glycolysis inhibitors such as dichloroacetate (DCA) to treat GBM tumors have yet to demonstrate any clear survival benefit for patients [9, 35]. The problems with treating tumors with anti-metabolic drugs arise when we consider the complex heterogeneity and plasticity of cancer cells. Heterogeneous tumor subpopulations may possess different energy and metabolic needs, therefore, targeting a single metabolic phenotype may not affect all subpopulations equally. Moreover, metabolic networks are highly interconnected and can be rewired to compensate for deficiencies in certain pathways [1, 20, 24]. In order to design better metabolism-based therapeutic strategies for the treatment of GBM tumors, metabolic preferences of distinct tumor subpopulations must be taken into consideration. Moreover, the effect of metabolism-targeting interventions on additional metabolic and growth-related compensatory processes must be assessed for the rational development of novel combinatorial therapies.

Cancer cells exist in a state of uncontrolled proliferation [16]. Induction of genotoxic or energetic stress can overwhelm cancer cells and lead to cell death. This is the guiding principle behind cancer-targeting therapies [16, 48]. If cancer-targeting agents cause irreparable damage, this can stimulate tumor suppressor pathways that will either activate a programmed cell death pathway, or can drive cells to exit the cell cycle and enter into a dormant, non-proliferative state known as senescence [10, 48]. The majority of cancer-targeting agents used in clinics are highly toxic and induce programmed cell death to stop tumor growth [48]. On the other side, senescence has also been deemed a desirable therapeutic outcome for cancer treatment as it has the potential to limit tumor cell proliferation without causing extensive damage to surrounding tissues from cell death and inflammatory signals [10]. However, the role of senescence in the response of heterogeneous tumor subpopulations to metabolism-targeting interventions is unclear. Autophagy is an intracellular catabolic process that can breakdown and recycle cellular constituents in response to nutrient deprivation [22, 70]. The role of autophagy in cancer is controversial, as it has been demonstrated that autophagy may act as a pro-survival or pro-death signal depending on the stimuli and stage of tumor development [67]. Recently, upregulation of autophagy has been shown to act as an adaptive resistance mechanism in response to chemotherapeutic stress in cancer cells [5, 25, 33, 57]. Hence, combination therapies targeting autophagy-mediated resistance mechanisms may represent an attractive therapeutic strategy to augment chemotherapy. Despite these insights, the role of autophagy in the response of heterogeneous tumor subpopulations to metabolic inhibitors remains unclear.

This study aims to investigate metabolic phenotypes of heterogeneous GBM tumor subpopulations and explore the role of cell-growth related processes, such as autophagy and senescence, in response to metabolism-targeting interventions. Using a clinically-relevant and patient-derived model of GBM, comprehensive metabolic profiling was performed on stem cell-like and non-stem-like tumor subpopulations. It was found that stem cell-like CD133/PROM1^HIGH^ patient-derived GBM cells possess enhanced levels of glycolytic enzymes and glycolytic activity as compared to their non-stem-like counterparts. Moreover, bioinformatic analysis demonstrated that glycolytic enzyme expression positively correlates with stemness markers in GBM patient tumor specimens. While treatment with glycolytic inhibitors induced senescence and inhibited cell growth in stem cell-like GBM subpopulations as compared to non-stem-like cells, blocking glycolysis alone did not hamper their stemness capacity. Further mechanistic analysis concluded that this was due to compensatory upregulation of autophagy. Reciprocally, inhibition of autophagy in stem cell-like tumor subpopulations corresponded with an increase in glycolytic activity, while also promoting senescence-related growth arrest without affecting stemness capacity. Combinatorial inhibition of glycolysis and autophagy led to a cumulative decrease in cell growth, driving cells towards apoptotic programmed cell death instead of senescence. This induction of cell death was supported by a significant impairment in cellular self-renewal capacity and expression of stemness-maintaining factors. These findings highlight the robust metabolic flexibility of stem cell-like tumor subpopulations and unveil an important mechanistic cross-talk between glycolysis, autophagy, and senescence that mediates a survival advantage under conditions of metabolic stress in heterogeneous GBM tumor subpopulations.

## Materials and methods

### Isolation and characterization of patient-derived glioblastoma tumor cells

The patient-derived glioblastoma (GBM) tumor cells were kindly provided by Dr. Sheila Singh (Stem Cell and Cancer Research Institute, McMaster University; MTA no. MTO20-120). Dr. Singh and colleagues performed the following isolation and characterization procedures of patient-derived samples as previously described [45]. Human GBM samples were obtained from consenting patients, as approved by the Hamilton Health Sciences/McMaster Health Sciences Research Ethics Board. Upon surgical removal, tumor tissues were dissociated in PBS containing 0.2 Wünsch unit/mL Liberase Blendzyme 3 (Roche) and incubated at 37 °C in a shaker for 15 min. The dissociated tissue was filtered through a 70 µm cell strainer and collected by centrifugation (450 g, 3 min). Red blood cells were lysed using ammonium chloride solution (STEMcell Technologies). The cells were washed with PBS and resuspended in NeuroCult^TM^ NS-A Proliferation Medium supplemented with 20 ng/mL EGF, 10 ng/mL bFGF, 2 µg/mL of Heparin and 1X antibiotic–antimycotic (Anti-Anti). The cells were then plated on ultra-low attachment plates (Corning) and propagated as neurospheres. An additional CD133/PROM1^HIGH^ patient-derived GBM cell line, GBM8, was provided as a kind gift from Dr. Hiroaki Wakimoto (Massachusetts General Hospital, Boston, MA, USA). Human astrocytes were purchased from ScienCell Research Laboratories, Carlsbad, CA, USA, and kindly provided by Dr. Donald Miller (University of Manitoba). Astrocytes were maintained in complete Astrocyte Medium (ScienCell Research Laboratories).

Neurospheres were dissociated into single cells and stained with APC-conjugated anti-CD133/PROM1 or a matched isotype control (Miltenyi) as recommended by the manufacturer and incubated for 15 min at room temperature. Samples were run on a MoFlo XDP Cell Sorter (Beckman Coulter). The expression of CD133/PROM1 was defined as positive or negative based on the analysis regions set on the isotype control.

### Cell culture and treatments

Patient-derived GBM cells were maintained in NeuroCult^TM^ NS-A Proliferation Medium supplemented with 20 ng/mL EGF, 10 ng/mL bFGF, 2 µg/mL of Heparin, and 1X Anti-Anti (NeuroCult complete media) and propagated as non-adherent neurospheres. Patient-derived cells were grown in 6 cm tissue culture plates at 37˚C in a humidified incubator containing 5% CO_**2**_. Patient-derived GBM neurospheres were passaged by manual dissociation of neurospheres into single cells through gentle trituration by pipetting.

Cells were treated with 2-DG (Sigma Aldrich) at the indicated doses for 24 h prior to analysis unless otherwise specified. Cells were treated with 10 µM of Spautin-1 (Selleckchem) for 24 h prior to analysis. For flux assays, cells were treated with 2-DG (1 mM) for 4 h and then treated with 12 µM of CQ 24 h prior to sample collection and analysis.

### Neurosphere formation assay

Patient-derived GBM cells were dissociated into single cell suspensions through gentle trituration by pipetting and cells were seeded at a low density of 1 × 10^3^ cells/mL in ultralow-attachment 6-well plates and maintained in NeuroCult complete media. Images of neurospheres were taken 5 days after initial seeding using a light microscope from multiple fields of view. Using ImageJ software (National Institutes of Health) for quantification of neurosphere images, spheres with a diameter equal to or larger than 50 µm were deemed neurospheres and the average number was determined from 3 independent experiments.

For the limiting dilution assay, patient-derived GBM cells were seeded at varying densities of 1, 5, 10, 25, 50, and 100 cells/well in multiple wells of a 96-well ultralow-attachment plate in NeuroCult complete media. After 5 days, wells were examined and the number of wells with neurospheres formed were counted. Limiting Dilution Analysis plot and calculations were generated using ELDA software from https://bioinf.wehi.edu.au/software/elda/.

### Protein extraction and western immunoblotting

Cell pellets were washed in cold 1X PBS at pH 7.4 and centrifuged at 500*g* for 5 min at 4 °C. Pellets were resuspended in RIPA lysis buffer (25 mM Tris pH 7.6, 150 mM NaCl, 1% NP-40, 1% sodium deoxycholate, 1% SDS) containing 1% PIC and phosphatase inhibitors. Whole cell lysates were incubated on ice for 45 min and then sonicated for 1 min. The samples were centrifuged at 20,000*g* for 15 min at 4 °C and the supernatants containing the proteins were collected. Protein concentrations were determined using the colorimetric Micro BCA^TM^ assay kit (Thermo Fisher) according to manufacturer’s instructions. Equal amounts of protein were boiled in Laemmli sample buffer (BioRad) containing 5% β-mercaptoethanol for 5 min and then resolved by SDS-PAGE. Protein was transferred onto nitrocellulose membranes (BioRad). Membranes were coated in Ponceau S dye to detect total protein concentration for normalization. Membranes were then washed in PBST to remove Ponceau S before blocking. Membranes were blocked in 5% non-fat milk in PBST (PBS, 0.05% Tween 20) at room temperature for 45 min, then washed in PBST and incubated in the appropriate primary antibody overnight at 4 °C with shaking. The primary antibodies were prepared at a 1:1000 dilution in 1% BSA in PBST. Details of specific antibodies used in this study can be found in Additional file 1: Table 1.

### Quantitative real-time PCR

RNA was extracted from patient-derived GBM cells using the Aurum total RNA Mini Kit (Bio-Rad) according to manufacturer’s protocol and cDNA was synthesized using iScript (Bio-Rad). Each sample of cDNA was quantitated and diluted to an equal concentration of 10 ng/mL. The Applied Biosystems 7300 real-time PCR machine was used for the quantitative real-time PCR (qRT-PCR), using SYBR Green Supermix (Bio-Rad). All primers, as described in Additional file 1: Table 2, were purchased from Invitrogen. *ACTB* was used for normalization of the genes of interest. The results were analyzed using 2^−ΔΔCT^ method and expressed as fold change to respective non-treated controls.

### Microscopy and imaging

For punctae formation, cells were seeded on coverslips 48 h before fixation at a density to be 70% confluent on the day of fixation. Cells were transfected with 4 µg/mL of *EGFP-MAP1LC3 *plasmid 24 h after seeding. Cells were subsequently treated with 12.5 µM of CQ 6 h after *EGFP-MAP1LC3* transfection. 48 h after seeding, cells were fixed using 4% paraformaldehyde. Following fixation, cells were washed in PBST and stained with 1 µg/mL DAPI for 1 min before coverslips were mounted to slides using Vectasheild mounting medium. Imaging was performed using Zeiss Axio Imager with Apoptome 2.

### Lactate assay

Growth media was collected 24 h post-treatment and lactate secretion was detected by using the lactate assay kit (Millipore Sigma, MAK064) following the manufacturer’s protocol. Absorbance was measured at 570 nm using a SpectraMax Plus plate reader.

### Glucose uptake

Cells were plated in 96-well Lumox plates (Sarstedt) in glucose-free DMEM media (ThermoFisher) with 150 µg/mL of 2-NBDG (Cayman Chemical) for 3 h. Media was removed and cells were washed in PBS and then lysed in 100µL of ice-cold lysis buffer containing 0.1 M potassium phosphate and 1% (v/v) Triton X-100 adjusted to pH 10 to release the fluorescent glucose analog (2-NBDG). The amount of 2-NBDG taken up by GBM cells in each well was quantified using a fluorometric plate reader (BioTek Synergy 2) using an excitation wavelength of 468 nm and an emission wavelength of 540 nm. A portion of the lysate (10 µL) was removed and analyzed for total protein content using the Micro BCA^TM^ assay kit and the fluorescent intensity was normalized to total protein concentration.

### Oroboros respirometry

Mitochondrial respiration was evaluated based on oxygen consumption using high resolution Oroboros oxygraphy (Oroboros Instruments GmbH, Innsbruck, Austria). In brief, an Oroboros oxygraph is a Clarke-type oxygen electrode that has two chambers (0.5 mL volume) equipped with oxygen sensors. Air calibration of these oxygen sensors is performed routinely on any day before starting a respirometric experiment.

To measure maximal oxygen consumption, patient-derived GBM cells were collected, counted, and 1 × 10^5^ cells were resuspended in serum-free complete NeuroCult media and added to the Oroboros oxygraphy chambers. Oxygen consumption rate (OCR) serves as a surrogate for mitochondrial electron transport chain function. OCR was measured at baseline and following sequential treatments with the ATP synthase inhibitor oligomycin, uncoupler carbonyl cyanide-p-(trifluoromethoxy) phenylhydrazone (FCCP) to remove the pH gradient and enable maximal rates of electron transport to occur, and antimycin A to block respiratory electron flux at Complex III. After measurement of basal respiration rates, the following chemicals were added: oligomycin (2 µM), FCCP (2–6 µM), and antimycin A (2 µM). The mitochondrial respiration parameters are defined as: Baseline respiration is termed basal respiration and maximal respiration is achieved by the addition of the uncoupler FCCP.

### β-Galactosidase/GBL1 assay

Senescence was detected by using the senescence β-Galactosidase/GLB1 staining kit (Cell Signaling) following the manufacturer’s protocol. The growth media was removed from the cells and the plate was washed one time with 1 × PBS (ThermoFisher). Cells were fixed with the 1 × Fixative solution and incubated for 15 min at room temperature. After the incubation, the plate was rinsed two times with 1X PBS and 1 mL of the β-Galactosidase/GLB1 Staining Solution was added to each plate. The plate was sealed with parafilm and incubated at 37 °C in a dry incubator. After 24 h, the images were captured using a light microscope.

### Bioinformatics analysis

Normalized data files for TCGA datasets (Nature GBM 2008 [6]) were downloaded from the cBioPortal for Cancer genomics [7, 13]. Correlations were performed between glycolysis enzymes (*PFKP, GAPDH, LDHB*, and *LDHA*) and stemness markers (CD133/*PROM1* and *SOX2*) and statistics (correlation index, *R,* and *p*-value) were calculated using Pearson correlation method.

### Statistical analysis

Statistical analysis was performed in GraphPad Prism 9. To assess significant differences between single measurements of two groups of normally distributed data, unpaired two-tailed student’s t-test was used. To assess significant differences between more than two groups of normally distributed data, we performed one-way or two-way analysis of variance (ANOVA), followed by either a Fishers Least Significant Difference (LSD) test or when all pairs of datasets were compared, Tukey’s multiple comparisons test was performed.

### Graphics and illustrations

Original graphics and schematics were generated using Microsoft PowerPoint and created with BioRender.com.

## Results

### Characterization of stem-cell populations in heterogeneous patient-derived GBM cells

**Fig. 1:**
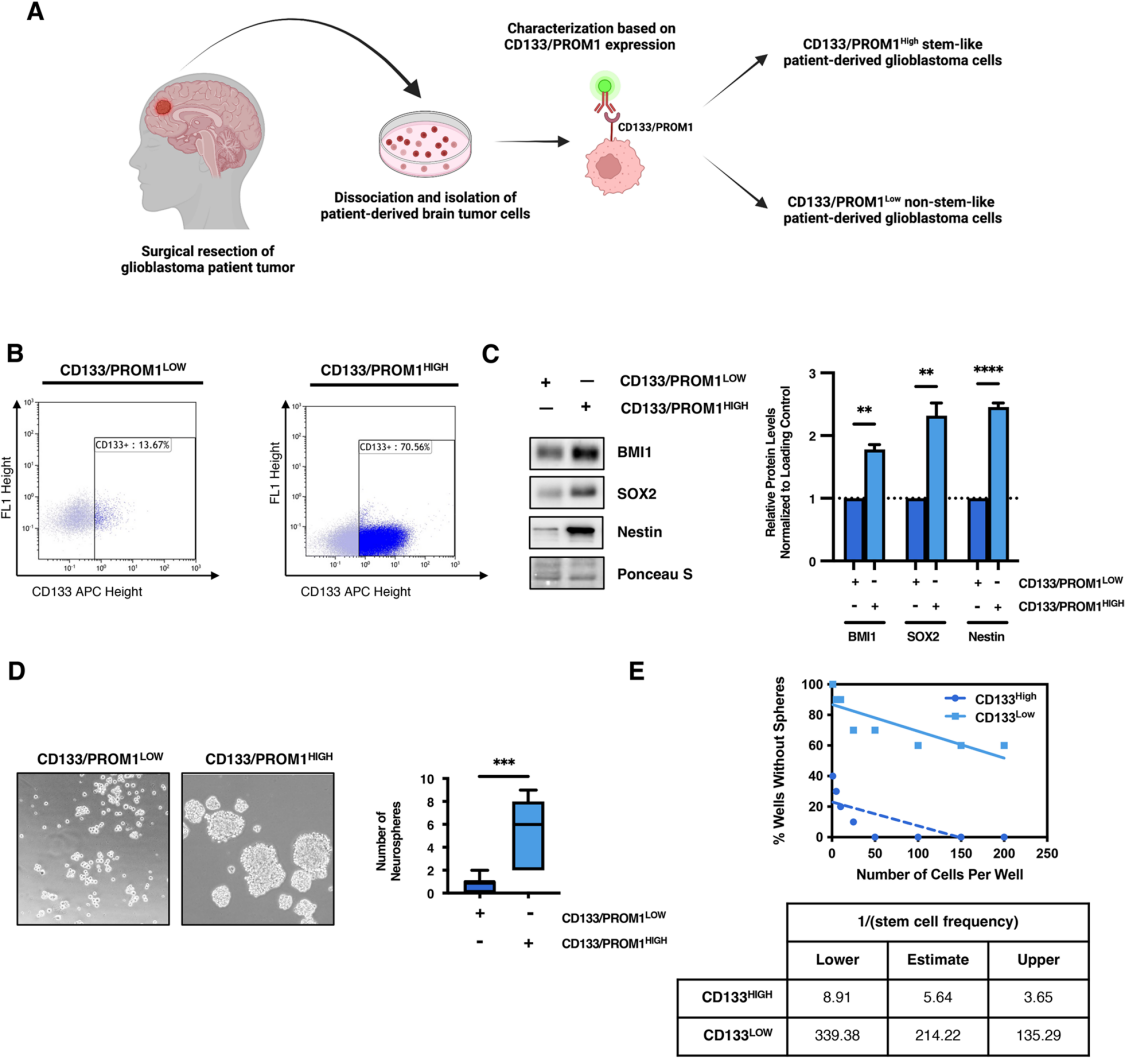
Heterogeneous tumor subpopulations possess different functional stemness capacity. **a** Schematic diagram depicting the isolation and characterization of patient-derived glioblastoma (GBM) cells. Created using Biorender.com. **b** Flow cytometry dot plots of CD133/PROM1 expression in CD133/PROM1^LOW^ and CD133/PROM1^HIGH^ patient-derived GBM cells. **c** Western blot analysis comparing the expression of BMI1, SOX2 and Nestin in CD133/PROM1^LOW^ and CD133/PROM1^HIGH^ patient-derived GBM cells and the graph represents densitometry quantification from three independent experiments. Statistical analysis was performed using two-sided students *t* test. *p < 0.05; **p < 0.01; ***p < 0.001; ns = non-significant. **d** CD133/PROM1^LOW^ and CD133/PROM1^HIGH^ patient-derived GBM cells were subjected to neurosphere formation analysis and the graph represents the quantification of neurospheres > 50 µm in size. Statistical analysis was performed using two-sided students t-test. *p < 0.05; **p < 0.01; ***p < 0.001; ns = non-significant. **e** Graph showing the proportion of wells without neurospheres formed in limiting dilution assay from CD133/PROM1^LOW^ and CD133/PROM1^HIGH^ patient-derived GBM cells. Table represents the Limiting Dilution Analysis calculations generated using ELDA software from https://bioinf.wehi.edu.au/software/elda/

To better understand the metabolic preferences of heterogeneous GBM tumors, we utilized clinically-relevant GBM cells derived from patient tumor specimens. To compare metabolic features of stem cell-like and non-stem-like GBM subpopulations, stem cell populations were quantified using the cell-surface stem cell marker CD133/PROM1 [51, 52] (Fig. 1a). While cell surface markers are commonly used to identify and isolate stem cell populations, they are not always the most reliable markers for functional stemness capacity when used alone [46, 47]. It is important to validate stemness characteristics using additional complementary methods. There are several well-established transcription factors that are important for maintaining the stemness of neural stem cells including but not limited to; Nestin/NES, SOX2, and BMI1 [49, 58, 62]. These factors are highly expressed in neural stem cells and progenitor cells, but their expression tends to be lost upon differentiation into mature neural lineages such as neurons and astrocytes [71]. Aberrant expression of these factors in cancer cells helps confer stemness characteristics and maintains stem cell-like tumor subpopulations [3, 4, 17, 58, 62]. Two distinct patient-derived heterogeneous GBM cell lines were characterized by flow cytometry into non-stem-like CD133/PROM1^LOW^ and stem cell-like CD133/PROM1^HIGH^ subpopulations (Fig. 1b; Additional file 1: Figure 1A). This was further validated by immunoblot analysis that confirmed CD133/PROM1^HIGH^ patient-derived GBM cells have higher overall protein expression of BMI1 (*p* < 0.01), SOX2 (*p* < 0.01), and Nestin (*p* < 0.0001) as well as mRNA levels of Nestin/*NES* (*p* < 0.001) and *SOX2* (*p* < 0.05) compared to CD133/PROM1^LOW^ cells (Fig. 1c; Additional file 1: Figure 1B). Comparison of self-renewal capacity by measuring neurosphere forming capacity confirmed that CD133/PROM1^HIGH^ patient-derived cell lines were enriched for self-renewing stem cell populations as compared to CD133/PROM1^LOW^ cells (*p* < 0.001; Fig. 1d). Additionally, limiting dilution assay (LDA) demonstrated that CD133/PROM1^HIGH^ patient-derived GBM cells had a higher proportion of self-renewing stem cell populations relative to CD133/PROM1^LOW^ cells (Fig. 1e; Additional file 1: Figure 1C). Altogether, these data allow us to distinguish between non-stem-like and stem cell-like patient-derived tumor subpopulations using both phenotypic and functional analysis in order to better understand the molecular and biochemical characteristics of heterogeneous patient tumors.

### Heterogeneous tumor subpopulations have unique metabolic genetic landscapes

**Fig. 2:**
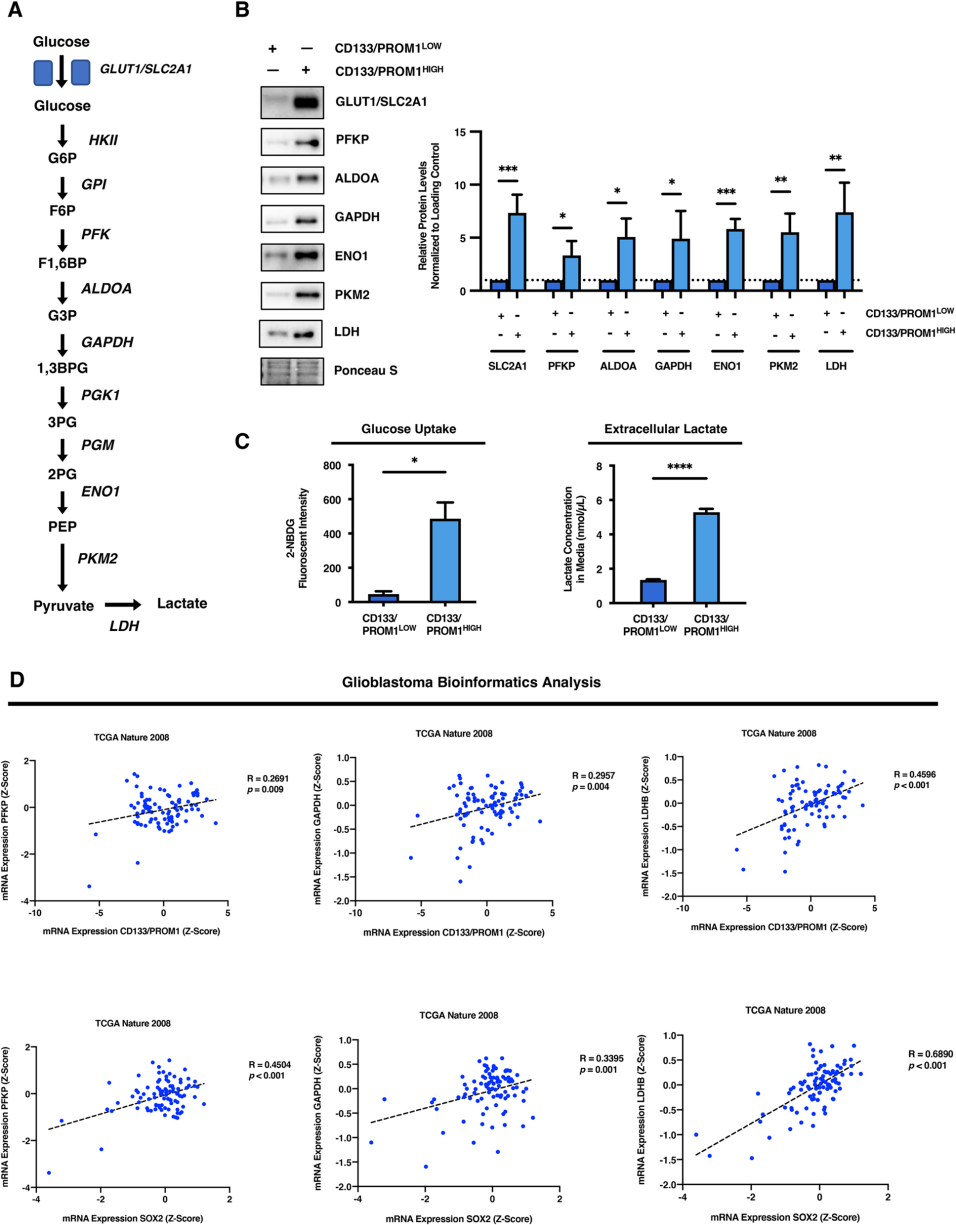
Heterogeneous tumor subpopulations differ in their glycolytic metabolic capacity. **a** Schematic diagram depicting the enzymes which catalyze the metabolic reactions that comprise the glycolytic pathway. **b** Western blot analysis comparing the expression of the glucose uptake receptor GLUT1/SLC2A1 and several glycolytic enzymes (PFKP, ALDOA, GAPDH, ENO1, PKM2, and LDH) in CD133/PROM1^LOW^ and CD133/PROM1^HIGH^ patient-derived GBM cells. Graph represents densitometry quantification of western blots from three independent experiments. Statistical analysis was performed using two-sided students t-test. *p < 0.05; **p < 0.01; ***p < 0.001; ns = non-significant. **c** Glucose uptake capacity was compared in CD133/PROM1^LOW^ and CD133/PROM1^HIGH^ patient-derived GBM cells by monitoring the accumulation of intracellular 2-NBDG through measuring the fluorescent intensity. Extracellular lactate levels were quantified in CD133/PROM1^LOW^ and CD133/PROM1^HIGH^ patient-derived GBM cells using a lactate assay and normalized to total protein concentration. Statistical analysis was performed using two-sided students t-test. *p < 0.05; **p < 0.01; ***p < 0.001; ns = non-significant. **d** Bioinformatic analysis of mRNA expression from 91 GBM biospecimens from the TGCA pilot (*Nature*, 2008). Pearson correlation between the expression of glycolytic enzymes (*PFKP, GAPDH*, and *LDHB*) and stemness markers (CD133/*PROM1* and *SOX2*) was performed and Pearson’s *r* correlation coefficients were calculated. Statistical analysis was performed using two-sided student’s t-test and exact *p *values are given.

After establishing functional differences between stem cell-like and non-stem-like heterogeneous GBM tumor subpopulations, we wished to determine whether these distinct cell types possess unique metabolic characteristics. Stem cell-like and non-stem-like patient-derived GBM tumor subpopulations were comprehensively screened for differences in the expression of enzymes involved in catalyzing the breakdown of glucose through the glycolytic pathway, including; the glucose transporter GLUT1/SLC2A1, phosphofructokinase (PFKP), aldolase A (ALDOA), glyceraldehyde-3-phosphate dehydrogenase (GAPDH), enolase 1 (ENO1), and pyruvate kinase isoform M2 (PKM2) (Fig. 2a, b). It was found that stem cell-like CD133/PROM1^HIGH^ patient-derived GBM cells harbored significantly higher basal levels of glycolytic enzymes compared to non-stem-like CD133/PROM1^LOW^ patient-derived cells (GLUT1/SLC2A1: *p* < 0.001, PFKP: *p* < 0.05, ALDOA: *p* < 0.05, GAPDH: *p* < 0.05, ENO1: *p* < 0.001; PKM2: *p* < 0.01; Fig. 2b). Elevated levels of glycolytic enzymes also corresponded with increased shuttling of pyruvate away from mitochondrial oxidative metabolism towards lactate production as indicated by increased expression of lactate dehydrogenase (LDH) in stem cell-like CD133/PROM1^HIGH^ compared to non-stem-like CD133/PROM1^LOW^ (LDH: *p* < 0.01; Fig. 2b). LDH is a multimeric complex consisting of four subunits comprised of different isoforms. The two main isoforms are LDHA and LDHB. We performed western blot analysis for specific LDH subunits and found that the protein abundance of both LDHA and LDHB was higher in stem cell-like CD133/PROM1^HIGH^ compared to non-stem-like CD133/PROM1^LOW^ (Additional file 1: Figure 2A).

To conclusively determine whether the overexpression of glycolytic enzymes in stem cell-like tumor subpopulations corresponds with enhanced glycolytic activity, glucose uptake capacity and lactate production of heterogeneous tumor subpopulations was evaluated. It was found that stem cell-like CD133/PROM1^HIGH^ patient-derived GBM cells displayed enhanced glucose uptake, as measured by intake of the fluorescent glucose analog 2-NBDG, as compared to non-stem-like CD133/PROM1^LOW^ patient-derived GBM cells (*p* < 0.05; Fig. 2c). Moreover, stem cell-like CD133/PROM1^HIGH^ GBM cells exhibited elevated LDH activity compared to their non-stem-like counterparts, as indicated by significantly increased lactate production and extracellular secretion (*p* < 0.0001, Fig. 2c).

The high glycolytic capacity demonstrated by stem cell-like CD133/PROM1^HIGH^ cells was attributed to significantly enhanced mRNA transcript levels of glycolytic enzymes (*ALDOA*: *p* < 0.05, *ENO1*: *p* < 0.05, *PKM2*: *p* < 0.01; *SLC2A1: p* < 0.05, *LDH*: *p* < 0.05), indicating that high expression of glycolytic enzymes may correspond with increased stemness in GBM tumors (Additional file 1: Figure 2B). This theory was confirmed using bioinformatic analysis of 91 GBM patient tumor samples from the TCGA Nature 2008 cohort [6]. It was found that high expression of the stemness markers *PROM1* or *SOX2* significantly correlates with high levels of several glycolytic enzymes (*GAPDH*, *LDHB* and *PFKP*) in GBM patient tumors (Fig. 2d). While the mRNA expression of *LDHA* was not significantly positivity correlated with stemness markers in patient GBM tumor samples (Additional file 1: Figure 2C), this can potentially be explained due to differences in protein abundance/activity that do not always correlate with mRNA levels. Furthermore, bulk tumor sequencing analyses are known to sometimes contain contaminating traces of normal tissues that could influence mRNA expression profiles [65]. Altogether, these findings highlight the unique metabolic activities of heterogeneous GBM tumor subpopulations and demonstrate a correlation between stemness and glycolysis gene expression signatures in GBM tumors.

Many cancer cells with enhanced glycolytic activity have been shown to display lower mitochondrial metabolic outputs [8, 31, 39]. As we observed that CD133/PROM1^HIGH^ patient-derived GBM cells demonstrated increased glycolytic function as compared to CD133/PROM1^LOW^ GBM cells, we further explored their differences in mitochondrial metabolic activity. Pyruvate generated through glycolysis can either be converted into lactate to help maintain ATP production independent of mitochondrial metabolism, or it can enter into the mitochondria where it is converted to acetyl-CoA by the action of pyruvate dehydrogenase (PDH) and is fed into the mitochondrial tricarboxylic acid cycle (TCA) to support ATP synthesis through oxidative phosphorylation in the electron transport chain (ETC) (Additional file 1: Figure 3A). Activity of PDH is tightly regulated by phosphorylation under the control of pyruvate dehydrogenase kinases (PDKs), which inhibit PDH activity [72] (Additional file 1: Figure 3A). We performed western blotting to compare the levels of the PDK1 isoform in CD133/PROM1^LOW^ versus CD133/PROM1^HIGH^ patient-derived GBM cells and found that PDK1 was higher in CD133/PROM1^HIGH^, suggesting inhibition of pyruvate-dependent mitochondrial metabolism (Additional file 1: Figure 3B). In line with this, we also found that the enzyme succinate dehydrogenase A (SDHA), which is involved in both the TCA cycle and the ETC, was lower in CD133/PROM1^HIGH^ patient-derived GBM cells compared to CD133/PROM1^LOW^ cells (Additional file 1: Figure 3B). Interestingly, we found no significant difference in the levels of the mitochondrial outer membrane protein TOM20, between CD133/PROM1^LOW^ and CD133/PROM1^HIGH^ patient-derived GBM cells, suggesting no major difference in the total mitochondrial content between these two subpopulations (Additional file 1: Figure 3B). Finally, to conclusively measure mitochondrial metabolic capacity in patient-derived GBM cells, we performed Oroboros respirometry to quantify the oxygen consumption rate (OCR) of CD133/PROM1^LOW^ and CD133/PROM1^HIGH^ patient-derived GBM cells. CD133/PROM1^HIGH^ patient-derived GBM cells had significantly lower basal respiration as well as maximal oxygen consumption capacity as compared to CD133/PROM1^LOW^ cells, indicating lower mitochondrial metabolic activity and respiration (Additional file 1: Figure 3C). Altogether, these findings demonstrate that the enhanced glycolytic activity exhibited by CD133/PROM1^HIGH^ patient-derived GBM cells corresponds with lower mitochondrial metabolism as compared to CD133/PROM1^LOW^ cells.

### Targeting glycolysis in stem cell-like tumor subpopulations hampers growth and promotes senescence but does not induce apoptosis

**Fig. 3:**
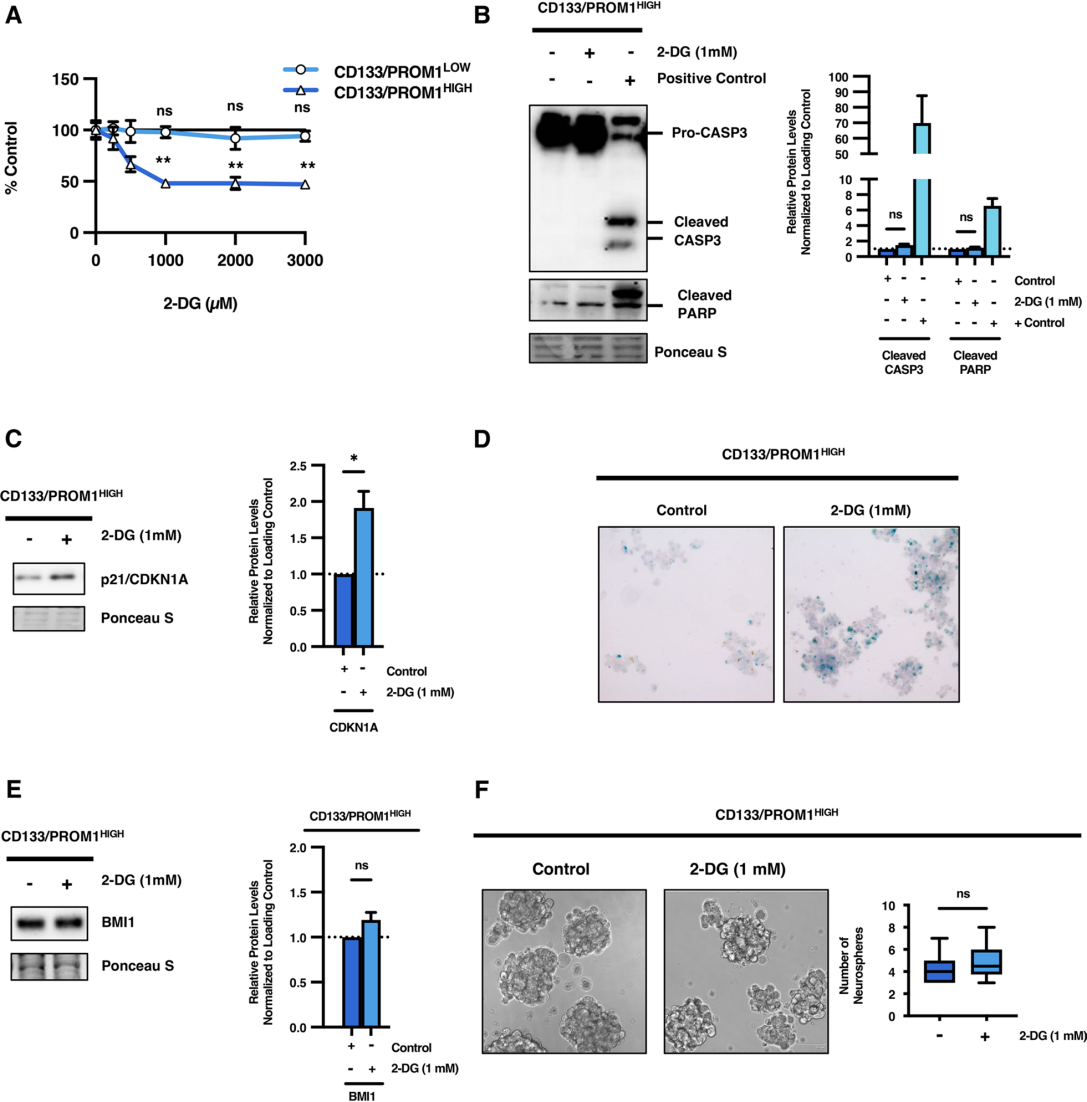
Glycolysis inhibition selectively impairs the growth of stem cell-like tumor subpopulations through induction of senescence but does not induce apoptosis or hamper stemness capacity. **a** CD133/PROM1^LOW^ and CD133/PROM1^HIGH^ patient-derived GBM cells were treated with increasing doses of 2-deoxyglucose (2-DG) from 0.25 to 3 mM. 24 h post-treatment cells were counted using trypan blue exclusion and then plotted as a percent of non-treated control cells. Statistical analysis was performed using two-sided students t-test. *p < 0.05; **p < 0.01; ***p < 0.001; ns = non-significant. **b** Western blot analysis of CD133/PROM1^HIGH^ patient-derived GBM cells following treatment with 1 mM of 2-DG for CASP3 (Pro-CASP3 35 kDa and cleaved CASP-3 17 & 19 kDa) and cleaved PARP (89 kDa). Positive control of cells treated with cytotoxic chemical were run in parallel. Graph represents densitometry quantification of western blots from three independent experiments. Statistical analysis was performed using two-sided students t-test. *p < 0.05; **p < 0.01; ***p < 0.001; ns = non-significant. **c** Western blot analysis of CD133/PROM1^HIGH^ patient-derived GBM cells following treatment with 1 mM of 2-DG for p21/CDKN1A expression. Graph represents densitometry quantification of western blots from three independent experiments. Statistical analysis was performed using two-sided students t-test. *p < 0.05; **p < 0.01; ***p < 0.001; ns = non-significant. **d** Non-treated controls and 2-DG (1 mM) treated CD133/PROM1^HIGH^ patient-derived GBM cells were subjected to β-galactosidase/GLB1 staining 24-h post-treatment to detect the presence of senescent cells. **e** Non-treated controls and 2-DG (1 mM) treated CD133/PROM1^HIGH^ patient-derived GBM cells were subjected to western blot analysis for the expression of BMI1. Graph represents densitometry quantification from three independent experiments. Statistical analysis was performed using two-sided students t-test. *p < 0.05; **p < 0.01; ***p < 0.001; ns = non-significant. **f** Non-treated controls and 2-DG (1 mM) treated CD133/PROM1^HIGH^ patient-derived GBM cells were subjected to neurosphere formation analysis and the graph represents the quantification of neurospheres > 50 µm in size. Statistical analysis was performed using two-sided students t-test. *p < 0.05; **p < 0.01; ***p < 0.001; ns = non-significant.

Given that it was observed that stem cell-like CD133/PROM1^HIGH^ patient-derived GBM cells possess high basal levels of glycolytic activity, we reasoned that these tumor subpopulations may be sensitive to glycolysis inhibition. Using the glucose analog 2-deoxyglucose (2-DG) to block glycolytic activity, we monitored the effect of dosing this competitive inhibitor on the growth of heterogeneous tumor subpopulations. A maximal decrease in cell number of ~ 50% was observed with treatment doses of 2-DG greater than 1 mM in stem cell-like CD133/PROM1^HIGH^ patient-derived GBM cells (1 mM: *p* < 0.01; 2 mM: *p* < 0.01; 3 mM: *p* < 0.01; Fig. 3a). We confirmed these findings using an additional glycolysis inhibitor, dichloroacetate (DCA), which inhibits PDK activity to shift glycolysis-associated lactate production towards mitochondrial metabolism. Similarly, we found that treatment with high dose of DCA (5 mM) led to a depletion in cell numbers of ~ 50% in CD133/PROM1^HIGH^ patient-derived GBM cells (*p* < 0.0001; Additional file 1: Figure 4A). In contrast, the growth of non-stem-like CD133/PROM1^LOW^ patient-derived GBM cells was not significantly affected by 2-DG treatment, even at doses up to 3 mM (Fig. 3a). However, 2-DG-induced suppression of cell growth in stem cell-like tumor subpopulations did not correspond with induction of apoptotic cell death, as indicated by a lack of caspase-3/CASP3 activation or downstream proteolytic cleavage of PARP in 2-DG treated CD133/PROM1^HIGH^ patient-derived GBM cells (CASP3 and PARP cleavage were detected using a positive control sample treated with cytotoxic chemotherapy) (Fig. 3b). Instead, it was found that glycolysis inhibition in stem cell-like CD133/PROM1^HIGH^ patient-derived GBM cells triggered induction of senescence-related cell cycle arrest, as indicated by significant upregulation in the protein and mRNA expression of p21^Waf1/Cip1^/*CDKN1A* and mRNA levels of p16^INK4A^/*CDKN2A* (Fig. 3c; Additional file 1: Figure 4B). Furthermore, β-galactosidase/GLB1 activity increased following glycolysis inhibition in stem cell-like tumor subpopulations, further corroborating an induction of senescence (Fig. 3d). These findings demonstrate a connection between cellular senescence and response of stem cell-like tumor subpopulations towards metabolic interventions.

### Stem cell-like tumor subpopulations maintain their stemness capacity following glycolysis inhibition

While slowing the growth of tumor cells seems to represent an attractive therapeutic outcome, it is not necessarily sufficient for curing cancer patients and preventing disease relapse. It has been demonstrated that many types of cancer stem cell-like populations display slower cycling rates and mostly exist in a dormant state of cellular senescence within tumors [36]. However, due to their stem cell-like nature, cancer stem cells have the capacity to re-enter the cell cycle and proliferate rapidly to promote tumor formation following therapy [26]. Hence, it is imperative to identify therapies which eliminate the stemness capacity of stem cell-like tumor subpopulations in order to prevent tumor recurrence. As such, the effect of 2-DG treatment on stemness properties of CD133/PROM1^HIGH^ patient-derived GBM cells was investigated. There was no significant change in the expression of stemness-maintaining factors following 2-DG treatment in stem cell-like tumor subpopulations (Fig. 3e; Additional file 1: Figure 4C). Furthermore, 2-DG treatment had no significant effect on the self-renewal capacity of CD133/PROM1^HIGH^ patient-derived GBM cells as measured by neurosphere formation ability (Fig. 3f). Altogether, these findings demonstrate that stem cell-like CD133/PROM1^HIGH^ patient-derived GBM possess enhanced glycolytic activity compared to their non-stem-like counterparts and targeting glycolysis can suppress the proliferation of stem cell-like GBM tumor subpopulations by promoting cellular senescence. However, inhibition of glycolysis alone does not induce apoptosis or suppress stemness in stem cell-like GBM tumor subpopulations, which indicates that these cells may activate alternative compensatory mechanisms to overcome metabolic stress.

### Glycolysis inhibition differentially regulates autophagy in heterogeneous tumor subpopulations

**Fig. 4:**
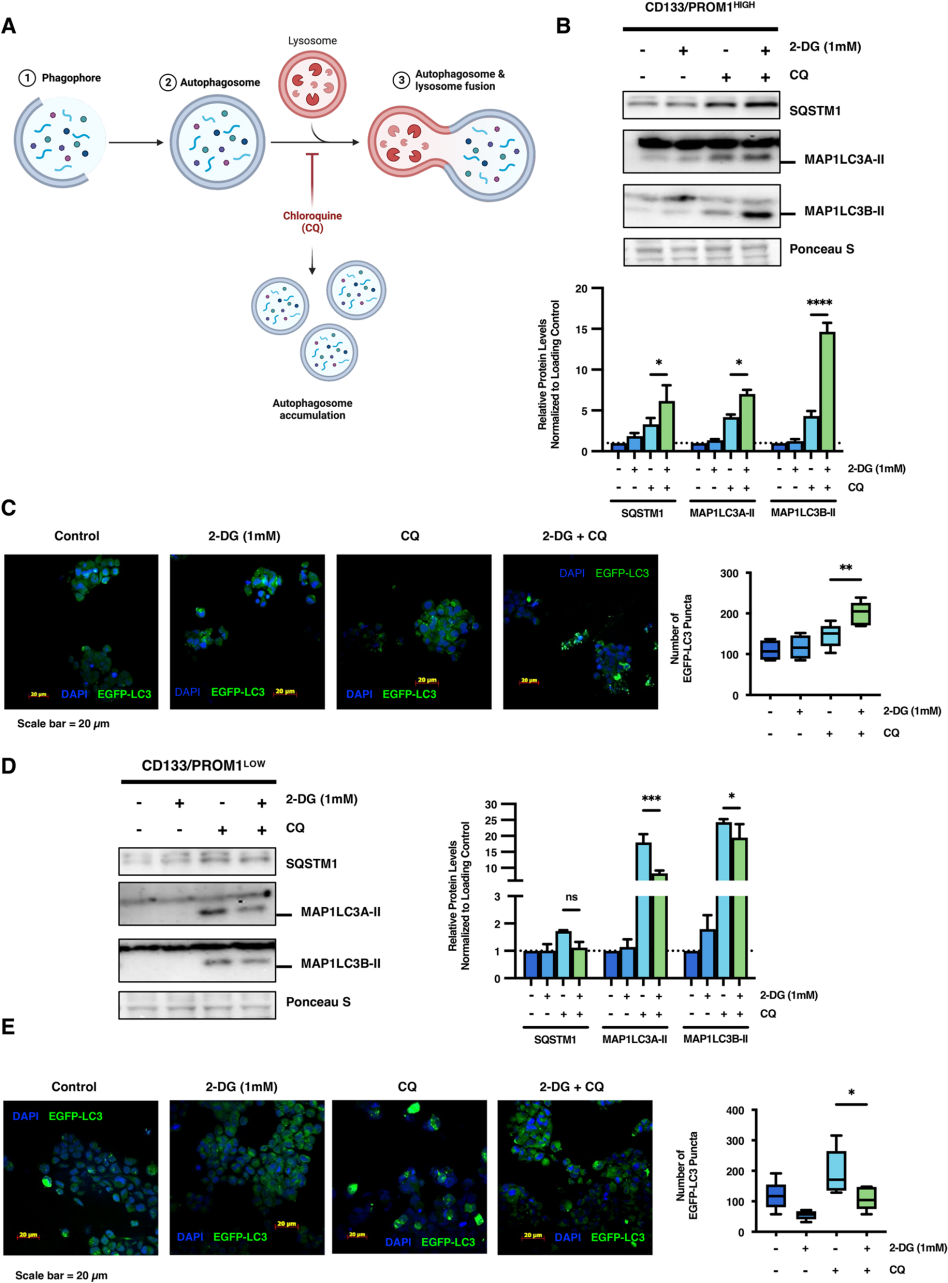
Glycolysis inhibition selectively induces autophagy in CD133/PROM1^HIGH^ stem cell-like patient-derived GBM cells but not CD133/PROM1^LOW^ non-stem-like patient-derived GBM cells.** a** Schematic diagram depicting the mechanism of action for chloroquine (CQ) and the principal of the autophagy flux assay. Created using Biorender.com. **b** CD133/PROM1^HIGH^ patient-derived GBM cells were treated with 2-DG (1 mM) and subjected to autophagy flux analysis where cells were treated with the late-stage autophagy inhibitor CQ and subjected to western blot analysis for the autophagosome-associated proteins SQSTM1, MAP1LC3A-II (14 kDa), and MAP1LC3B-II (14 kDa). Graph represents densitometry quantification of western blots from at least 3 independent experiments. Statistical analysis was performed using ANOVA. *p < 0.05; **p < 0.01; ***p < 0.001; ns = non-significant**. c** CD133/PROM1^HIGH^ patient-derived GBM cells with exogenous overexpression of EGFP-LC3 were treated with 2-DG (1 mM) and/or the late-stage autophagy inhibitor CQ and autophagosome assembly was assessed by monitoring the formation of EGFP-LC3^+^ punctae and the number of EGFP-LC3^+^ punctae were quantified. Statistical analysis was performed using ANOVA. *p < 0.05; **p < 0.01; ***p < 0.001; ns = non-significant. **d** CD133/PROM1^LOW^ patient-derived GBM cells were treated with 2-DG (1 mM) and subjected to autophagy flux analysis where cells were treated with the late-stage autophagy inhibitor CQ and subjected to western blot analysis for the autophagosome-associated proteins SQSTM1, MAP1LC3A-II (14 kDa), and MAP1LC3B-II (14 kDa). Graph represents densitometry quantification of western blots from at least 3 independent experiments. Statistical analysis was performed using ANOVA. *p < 0.05; **p < 0.01; ***p < 0.001; ns = non-significant. **e** CD133/PROM1^LOW^ patient-derived GBM cells with exogenous overexpression of EGFP-LC3 were treated with 2-DG (1 mM) and/or the late-stage autophagy inhibitor CQ and autophagosome assembly was assessed by monitoring the formation of EGFP-LC3^+^ punctae and the number of EGFP-LC3^+^ punctae were quantified. Statistical analysis was performed using ANOVA. *p < 0.05; **p < 0.01; ***p < 0.001; ns = non-significant

Autophagy is a metabolism-responsive catabolic process that can break-down and recycle cellular constituents to maintain energy requirements [37]. Induction of autophagy has been shown to mediate resistance to therapeutic stress in various cancer cells [5, 25]. Moreover, autophagy has been demonstrated to play an important role in maintaining stemness of normal and cancerous stem cells [14, 18, 43, 50]. Therefore, we postulated that autophagy may be modulated in response to metabolic stress caused by glycolysis inhibition in stem cell-like tumor subpopulations. To monitor autophagic activity in heterogeneous tumor subpopulations, we assessed the effect of 2-DG treatment on autophagy flux using two different techniques. First, we measured autophagy flux by immunoblot analysis for the autophagosome-associated proteins p62/SQSTM1 and MAP1LC3A/B-II. We measured expression of these autophagosome-associated proteins following 2-DG treatment in the presence or absence of late-stage autophagy inhibitor chloroquine (CQ). CQ inhibits the fusion of the autophagosome with the lysosome, thereby inhibiting the degradation of autophagosome-associated proteins [27] (Fig. 4a). This assay monitors flux of active autophagic degradation of proteins as opposed to changes in steady-state expression of autophagosome proteins [27]. It was found that treatment of CD133/PROM1^HIGH^ patient-derived GBM cells with 2-DG significantly enhanced accumulation of p62/SQSTM1 (*p* < 0.05), MAP1LC3A-II (*p* < 0.05), an MAP1LC3B-II (*p* < 0.0001) following treatment with CQ compared to control cells treated with CQ, indicating elevated autophagic flux in 2-DG-treated cells (Fig. 4b). Furthermore, an additional complementary method to examine autophagosome assembly was employed by exogenously expressing *EGFP-MAP1LC3B* [27]. When autophagy is activated, EGFP-MAP1LC3B is incorporated into the autophagosome membranes and autophagosome assembly can be monitored by measuring the formation of EGFP-MAP1LC3B^+^ punctae in the presence or absence of CQ [27]. We found that inhibition of autophagosome degradation using CQ treatment alone led to the accumulation of EGFP-MAP1LC3B^+^ punctae in CD133/PROM1^HIGH^ patient-derived GBM cells. Moreover, inhibition of glycolysis using 2-DG following CQ treatment further enhanced the accumulation of EGFP-MAP1LC3 punctae (*p* < 0.01), further supporting the notion that glycolysis inhibition promotes autophagy flux in stem cell-like CD133/PROM1^HIGH^ cells (Fig. 4c). These findings indicate that upregulation of autophagy following glycolysis inhibition may act as a compensatory response to mitigate metabolic stress in CD133/PROM1^HIGH^ patient-derived GBM cells.

After observing the strong induction of autophagy following glycolysis inhibition in stem cell-like CD133/PROM1^HIGH^ tumor subpopulations, we were interested to determine whether this was a specific response in cells that rely on high glycolytic activity, or a universal phenomenon that occurs across heterogeneous tumor subpopulations. Therefore, we assessed effect of 2-DG treatment on regulation of autophagy in non-stem-like CD133/PROM1^LOW^ tumor subpopulations. In contrast to observations in CD133/PROM1^HIGH^ cells, autophagy flux was not upregulated in 2-DG treated CD133/PROM1^LOW^ cells using both immunoblot analysis and EGFP-MAP1LC3^+^ puncta formation assay following CQ treatment (Fig. 4d, e) [27]. It was found that CQ did not increase the expression of autophagosome-associated proteins SQSTM1 and MAP1LC3A/B-II in 2-DG treated cells compared to controls (Fig. 4d). Moreover, the number of EGFP-MAP1LC3^+^ punctae was not significantly increased following 2-DG treatment in CD133/PROM1^LOW^ cells, and punctae did not accumulate in 2-DG treated cells combined with CQ as compared to controls (Fig. 4e).

Altogether, these findings demonstrate that autophagy is differentially regulated in response to glycolysis inhibition in heterogeneous tumor subpopulations. This indicates that autophagy upregulation may act as a compensatory response to mitigate metabolic stress in stem cell-like tumor subpopulations with high basal levels of glycolytic activity.

### Cross-talk between autophagy and glycolysis compensates during metabolic stress and confers metabolic plasticity in stem cell-like GBM tumor subpopulations

**Fig. 5:**
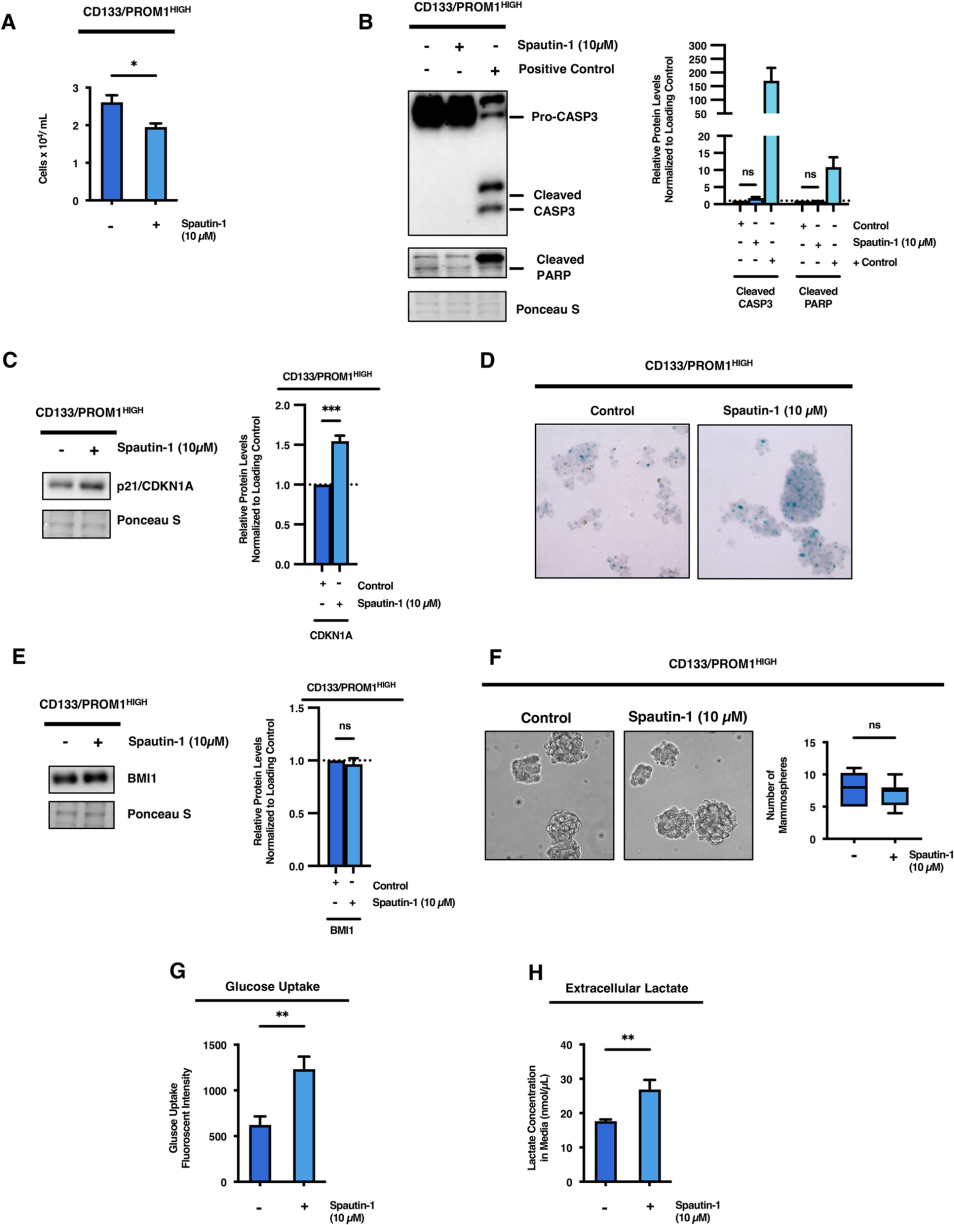
Autophagy inhibition reciprocally promotes autophagy in stem cell-like tumor subpopulations and regulates senescence to maintain stemness.** a** CD133/PROM1^HIGH^ patient-derived GBM cells were treated with 10 µM of Spautin-1 and the number of cells were counted after 24 h using trypan blue exclusion. Statistical analysis was performed using two-sided students t-test. *p < 0.05; **p < 0.01; ***p < 0.001; ns = non-significant. **b** Western blot analysis of CD133/PROM1^HIGH^ patient-derived GBM cells following treatment with 10 µM of Spautin-1 for CASP3 (Pro-CASP 35 kDa and cleaved CASP-3 17 & 19 kDa) and cleaved PARP (89 kDa). Positive control of cells treated with cytotoxic chemical were run in parallel. Graph represents densitometry quantification of western blots from three independent experiments. Statistical analysis was performed using two-sided students t-test. *p < 0.05; **p < 0.01; ***p < 0.001; ns = non-significant. **c** Western blot analysis of CD133/PROM1^HIGH^ patient-derived GBM cells following treatment with 10 µM of Spautin-1 for p21/CDKN1A expression. Graph represents densitometry quantification of western blots from three independent experiments. Statistical analysis was performed using two-sided students t-test. *p < 0.05; **p < 0.01; ***p < 0.001; ns = non-significant. **d** Non-treated controls and Spautin-1 (10 µM) treated CD133/PROM1^HIGH^ patient-derived GBM cells were subjected to β-galactosidase/GLB1 staining 24-h post-treatment to detect the presence of senescent cells. **e** Non-treated controls and Spautin-1 (10 µM) treated CD133/PROM1^HIGH^ patient-derived GBM cells were subjected to western blot analysis for the expression of BMI1. Graph represents densitometry quantification from three independent experiments. Statistical analysis was performed using two-sided students t-test. *p < 0.05; **p < 0.01; ***p < 0.001; ns = non-significant. **f** Non-treated controls and Spautin-1 (10 µM) treated CD133/PROM1^HIGH^ patient-derived GBM cells were subjected to neurosphere formation analysis and the graph represents the quantification of neurospheres > 50 µm in size. Statistical analysis was performed using two-sided students t-test. *p < 0.05; **p < 0.01; ***p < 0.001; ns = non-significant. **g** Non-treated controls and Spautin-1 (10 µM) treated CD133/PROM1^HIGH^ patient-derived GBM cells were subjected to glucose uptake assay and the fluorescent intensity of intracellular 2-NBDG was quantified. Statistical analysis was performed using two-sided students t-test. *p < 0.05; **p < 0.01; ***p < 0.001; ns = non-significant. **h** Extracellular lactate levels were quantified in non-treated controls and Spautin-1 (10 µM) treated CD133/PROM1^HIGH^ patient-derived GBM cells using a lactate assay and normalized to total protein concentration. Statistical analysis was performed using two-sided students t-test. *p < 0.05; **p < 0.01; ***p < 0.001; ns = non-significant

After observing that autophagy is selectively upregulated following 2-DG treatment in stem cell-like tumor subpopulations with high basal glycolytic activity, we wished to further characterize the role of autophagy in maintaining stemness and growth of CD133/PROM1^HIGH^ patient-derived cells. For this purpose, the upstream autophagy inhibitor, Spautin-1, was utilized [30]. Spautin-1 inhibits deubiquitinating enzymes USP10 and USP13 and promotes degradation of the BECN1-PI3K autophagy initiation complex, which is required for canonical autophagy induction [30]. Following treatment with Spautin-1 alone in stem cell-like CD133/PROM1^HIGH^ patient-derived cells, we observed a significant decrease in cell proliferation (*p* < 0.05) without inducing apoptotic cell death (Fig. 5a, b). Similar to glycolysis inhibition, this decrease in cell growth induced by blocking autophagy was accompanied by induction of senescence as indicated by significantly enhanced expression of p21^Waf1/Cip1^/*CDKN1A* and p16^INK4A^/*CDKN2A* as well as increased β-galactosidase/GLB1 activity (Fig. 5c, d; Additional file 1: Figure 5A). Moreover, autophagy inhibition by Spautin-1 had no significant effect on the stemness properties of stem cell-like CD133/PROM1^HIGH^ GBM cells (Fig. 5e, f; Additional file 1: Figure 5B). Interestingly, we found that autophagy inhibition was accompanied by an increase in glycolytic activity as demonstrated by significantly enhanced glucose uptake capacity (*p* < 0.01) and increased lactate production (*p* < 0.01; Fig. 5g, h). These findings demonstrate a metabolic cross-talk between glycolysis, autophagy, and senescence in stem cell-like GBM tumor subpopulations and suggest that metabolic plasticity may promote cell survival during metabolic stress through senescence-induced cell cycle arrest.

### Concomitant inhibition of glycolysis and autophagy drives a switch from senescence towards cell death in stem cell-like GBM tumor subpopulations

**Fig. 6:**
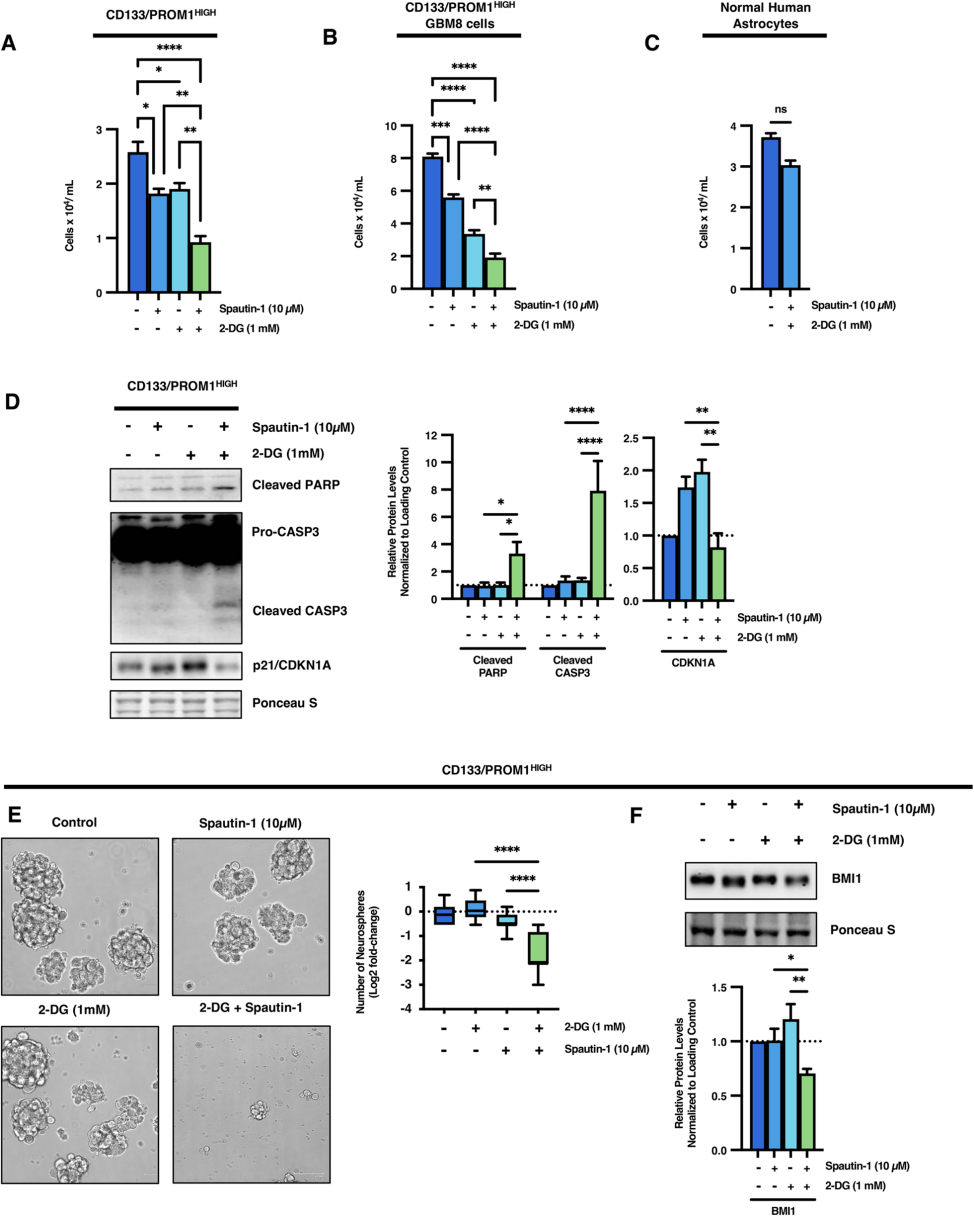
Combination of glycolysis and autophagy inhibition cumulatively decreases the growth of stem cell-like tumor subpopulations and suppresses stemness by blocking induction of senescence and promoting apoptosis. **a** CD133/PROM1^HIGH^ patient-derived GBM cells were treated with 2-DG (1 mM) and/or the autophagy inhibitor Spautin-1 (10 µM) for 24 h and the number of viable cells were counted using trypan blue exclusion. Statistical analysis was performed using ANOVA. *p < 0.05; **p < 0.01; ***p < 0.001. **b** An additional independent CD133/PROM1^HIGH^ patient-derived GBM cell line, GBM8, was treated with 2-DG (1 mM) and/or the autophagy inhibitor Spautin-1 (10 µM) for 24 h and the number of viable cells were counted using trypan blue exclusion. Statistical analysis was performed using ANOVA. *p < 0.05; **p < 0.01; ***p < 0.001. **c** Normal human astrocytes were treated with a combination of 2-DG (1 mM) and the autophagy inhibitor Spautin-1 (10 µM) for 24 h and the number of viable cells were counted using trypan blue exclusion. Statistical analysis was performed using two-sided students t-test. *p < 0.05; **p < 0.01; ***p < 0.001. **d** CD133/PROM1^HIGH^ patient-derived GBM cells were treated with 2-DG (1 mM) and/or Spautin-1 (10 µM) and subjected to western blot analysis for CASP3 (Pro-CASP 35 kDa and cleaved CASP-3 17 & 19 kDa), cleaved PARP (89 kDa), and p21/CDKN1A. Graph represents densitometry quantification from three independent experiments. Statistical analysis was performed using ANOVA. *p < 0.05; **p < 0.01; ***p < 0.001; ns = non-significant.** e** CD133/PROM1^HIGH^ patient-derived GBM cells were treated with 2-DG (1 mM) and/or Spautin-1 (10 µM) and subjected to neurosphere formation analysis and the graph represents the quantification of neurospheres > 50 µm. Statistical analysis was performed using ANOVA. *p < 0.05; **p < 0.01; ***p < 0.001 ns = non-significant. **f** CD133/PROM1^HIGH^ patient-derived GBM cells were treated with 2-DG (1 mM) and/or Spautin-1 (10 µM) and subjected to western blot analysis for the expression of BMI1. Graph represents densitometry quantification from three independent experiments. Statistical analysis was performed using ANOVA. *p < 0.05; **p < 0.01; ***p < 0.001; ns = non-significant

**Fig. 7:**
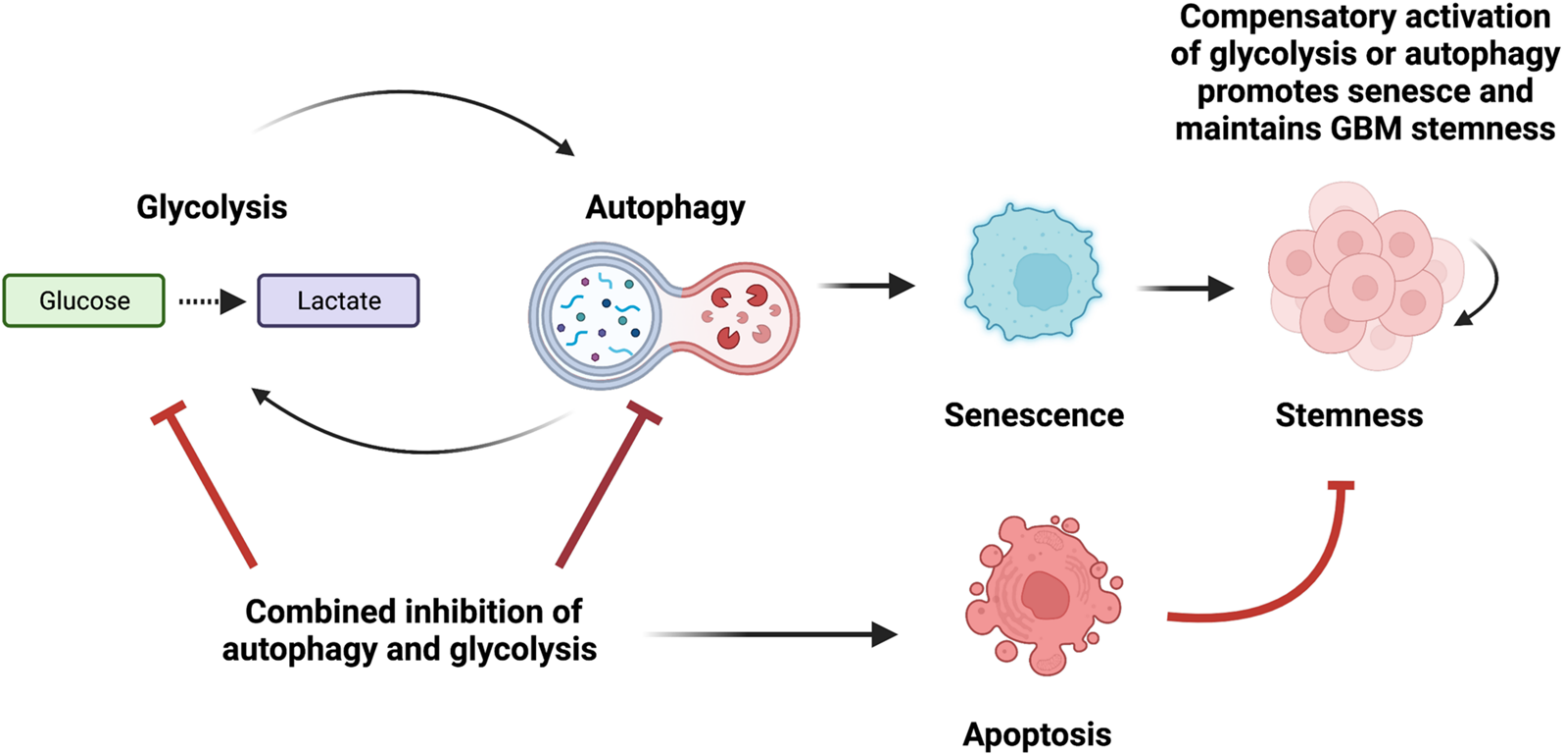
Glycolysis, autophagy, and senescence engage in a cross-talk that helps maintain the stemness capacity of GBM tumor subpopulations. Schematic diagram summarizing the role of compensatory cross-talk between glycolysis and autophagy on the fate of stem cell-like GBM tumor subpopulations. Created with Biorender.com

Our findings thus far demonstrate a clear cross-talk between glycolysis, autophagy, and senescence in stem cell-like tumor subpopulations. Moving forward, we wished to conclusively determine whether these two processes compensate for each other and whether this metabolic plasticity acts as a pro-survival adaptive mechanism during metabolic stress in stem cell-like brain tumor subpopulations. It was found that combinatorial treatment with Spautin-1 and 2-DG led to a significantly greater growth inhibition of CD133/PROM1^HIGH^ patient-derived GBM cells than either monotherapy (Fig. 6a). We confirmed these findings using an additional well-characterized CD133/PROM1^HIGH^ patient-derived GBM cell line, GBM8, which has previously been demonstrated to contain > 90% CD133/PROM1^HIGH^ cells [63, 64]. While both 2-DG and Spautin-1 impaired the cell numbers of GBM8 cells alone, the combinatorial treatment was significantly more effective (Fig. 6b). Importantly, this combination treatment showed no significant toxicity towards normal human astrocyte glial cells (Fig. 6c). Moreover, while neither Spautin-1 or 2-DG treatment induced apoptosis alone, combination treatment led to robust activation of apoptosis, as indicated by activation and cleavage of CASP3 as well as it’s downstream target PARP in stem cell-like tumor subpopulations (Fig. 6d). This switch towards apoptotic cell death also suppressed the induction of cellular senescence in stem cell-like GBM tumor subpopulations, where the upregulation of p21^Waf1/Cip1^/CDKN1A was blocked in 2-DG treated cells combined with Spautin-1 (Fig. 6d). These findings suggest that combination of autophagy and glycolysis inhibition may represent an effective strategy for the elimination of stem cell-like tumor subpopulations through induction of apoptosis as opposed to pushing them towards a dormant senescent state where they can later re-enter the cell cycle and re-initiate tumor formation. To further determine whether this combination strategy can suppress the aggressive properties of stem cell-like tumor subpopulations responsible for tumor recurrence, the effect of Spautin-1 and 2-DG combination treatment on stemness of CD133/PROM1^HIGH^ patient-derived GBM cells was assessed. While neither Spautin-1 or 2-DG treatment alone affected the self-renewal capacity of stem cell-like GBM tumor subpopulations, combinatorial therapy significantly inhibited neurosphere formation capacity (*p* < 0.0001; Fig. 6e). This decrease in stemness capacity was also accompanied by a significant downregulation of the functional stemness factor BMI-1 (Fig. 6f). Altogether, these findings highlight the metabolic cross-talk between glycolysis, autophagy and senescence in maintaining stemness and survival of stem cell-like GBM tumor subpopulations and demonstrate combinatorial inhibition of these processes as an attractive therapeutic strategy, as summarized in Fig. 7.

## Discussion

Cancer researchers and clinicians have been trying to leverage the enhanced energy requirements of tumor cells for therapeutic purposes for many years [32]. Despite their unique metabolic preferences, targeting metabolism, specifically targeting the Warburg effect and blocking glycolysis, has been largely unsuccessful in clinical practice [60]. To understand the reasons behind failure of metabolism-targeting therapies, it is important to consider the complex metabolic heterogeneity and plasticity of tumor subpopulations [24, 38]. Moreover, cross-talk between different metabolic pathways and non-metabolic energy responsive pathways in regulating cell survival is still poorly understood. Here, we dissect metabolic profiles between non-stem-like and stem cell-like tumor subpopulations from patient-derived GBM cells. Previous studies have reported differences in the metabolic phenotypes of stem cell-like GBM tumor-initiating populations *versus* their non-stem-like counterparts, however, there have been some contradicting findings [61, 73]. Some studies have demonstrated that stem cell-like populations exhibit increased glycolytic capacity compared to their differentiated counterparts, while others suggest they are more reliant on mitochondrial oxidative phosphorylation [61, 73]. The majority of these investigations have utilized established GBM cell line models that have undergone transformation and long-term culture which may not accurately reflect physiological conditions of patient tumors [54]. Our investigations determined that stem cell-like patient-derived tumor subpopulations characterized based on expressional and functional analysis display increased basal glycolytic activity and lower mitochondrial respiration as compared to non-stem-like patient-derived GBM cells. These findings were supported by bioinformatic analysis which demonstrated that the expression of stemness markers positively and significantly correlates with the expression of several glycolytic enzymes in GBM patient tumor datasets.

Not surprisingly, the high glycolytic capacity of stem cell-like tumor subpopulations rendered them more sensitive to growth inhibition by glycolysis inhibitors as compared to non-stem-like tumor populations. These findings are in line with previous investigations in cancers that have shown promising in vitro findings utilizing glycolysis inhibitors to suppress GBM cell growth [35, 73]. However, the effect of glycolysis inhibitors alone on aggressive properties of stem cell-like tumor subpopulations has not been well characterized in brain cancers. While we found that 2-DG treatment promoted senescence in patient-derived GBM stem-like cells, inhibition of glycolysis alone was insufficient to hamper their aggressive stemness properties or induce apoptotic cell death. Mechanistic analysis unveiled that this resilience towards metabolic stress displayed by stem cell-like tumor subpopulations is, at least in part, due to compensatory upregulation of autophagy flux. Furthermore, we uncovered a novel two-way communication between glycolysis and autophagy that regulates senescence and maintains stemness. We found that inhibition of autophagy alone also hampered the growth of stem cell-like tumor subpopulations and induced senescence without affecting stemness capacity, and this corresponded with a significant increase in glycolytic activity. Suppression of autophagy through genetic modulation has previously been demonstrated to hamper GBM development and promote cellular senescence [12], however, our findings suggest that induction of senescence may act as pro-survival mechanism that allows stem cell-like tumor subpopulations to persist during metabolic stress. While many GBM tumors carry deletions in the p16^INK4A^/CDKN2A gene [59], here we assessed various markers to measure the induction of senescence following glycolysis/autophagy inhibition, and we found that p21^Waf1/Cip1^/CDKN1A (which is not frequently lost or mutated in GBM tumors) is upregulated in addition to p16^INK4A^/CDKN2A. This suggests that induction of senescence may be mediated by both p16^INK4A^/CDKN2A and p21^Waf1/Cip1^/CDKN1A. Future studies exploring the in-depth role of p21^Waf1/Cip1^/CDKN1A in mediating this interplay between glycolysis, autophagy, and senescence would be of interest to better understand the clinical implications for precision medicine.

Here, we demonstrate that co-targeting glycolysis and autophagy by using Spautin-1 and 2-DG significantly suppressed the growth of stem cell-like tumor subpopulations compared to treatment with either therapy alone. Moreover, combinatorial treatment with Spautin-1 and 2-DG repressed the induction of senescence in stem cell-like tumor subpopulations which abrogated their self-renewal capacity and stemness and drove cells towards apoptotic cell death. Future studies investigating the therapeutic potential of such a combination strategy using in vivo animal xenograft models will be critical for future clinical translation. This would be especially important since some glycolytic inhibitors such as DCA are already entering into phase II clinical trials for GBM (NCT05120284), and the identification of autophagy as a potential targetable resistance mechanism opens up a therapeutic strategy to improve treatment responses for patients.

## Conclusions

The findings from this study provide novel and important insights regarding the robust flexibility and adaptability of stem cell-like tumor subpopulations to therapeutic and energetic perturbations. These findings shed light on the compensatory cross-talk that occurs between the major energy producing pathway glycolysis and the cellular degradation process autophagy which may be a reason behind the clinical failure of glycolysis inhibitors in patient trials. Moreover, this study highlights the need to exercise caution when evaluating novel therapies using suppression of cell growth and induction of senescence as readouts of efficacy, as our data demonstrate that that this does not necessarily effectively eliminate aggressive tumor-seeding populations. Altogether, the findings from this study unveil a novel mechanistic cross-talk between glycolysis, autophagy, and senescence in stem cell-like tumor subpopulations that has clinical implications to consider when devising therapeutic strategies to effectively eliminate all tumor subpopulations within heterogeneous tumors.

## Supplementary Information

**Additional file 1** Supplementary Figures and Tables.

## Data Availability

All data generated or analysed during this study are included in this manuscript (and its Additional file 1). The TCGA GBM patient data used in this study are available from BioPortal for Cancer Genomics [7, 13, 19] https://www.cbioportal.org/.

## Abbreviations

2-DG: 2-Deoxyglucose
ALDOA: Aldolase A
CASP3: Caspase-3
CQ: Chloroquine
DCA: Dichloroacetate
ENO1: Enolase 1
GAPDH: Glyceraldehyde-3-phosphate dehydrogenase
GBM: Glioblastoma
GPI: Glucose-6-phosphate isomerase
HK: Hexokinase
LDH: Lactate dehydrogenase
MAP1LC3: Microtubule-associated protein 1 light chain 3
PDK1: Pyruvate dehydrogenase kinase 1
PFKP: Phosphofructose kinase
PGK1: Phosphoglycerate kinase 1
PKM2: Pyruvate kinase M2
SDHA: Succinate dehydrogenase a
SQSTM1: P62/sequestome 1
