# Supplementary material for: Compensatory cross-talk between autophagy and glycolysis regulates senescence and stemness in heterogeneous glioblastoma tumor subpopulations
Source: Acta Neuropathol Commun. 2023 Jul 7;11:110. doi: 10.1186/s40478-023-01604-y (PMC10327182; doi:10.1186/s40478-023-01604-y)
Supplement: Supplementary file 1 — Additional file 1 Supplementary Figures and Tables. [file 40478_2023_1604_MOESM1_ESM.pdf]

SUPPLEMENTARY FIGURES

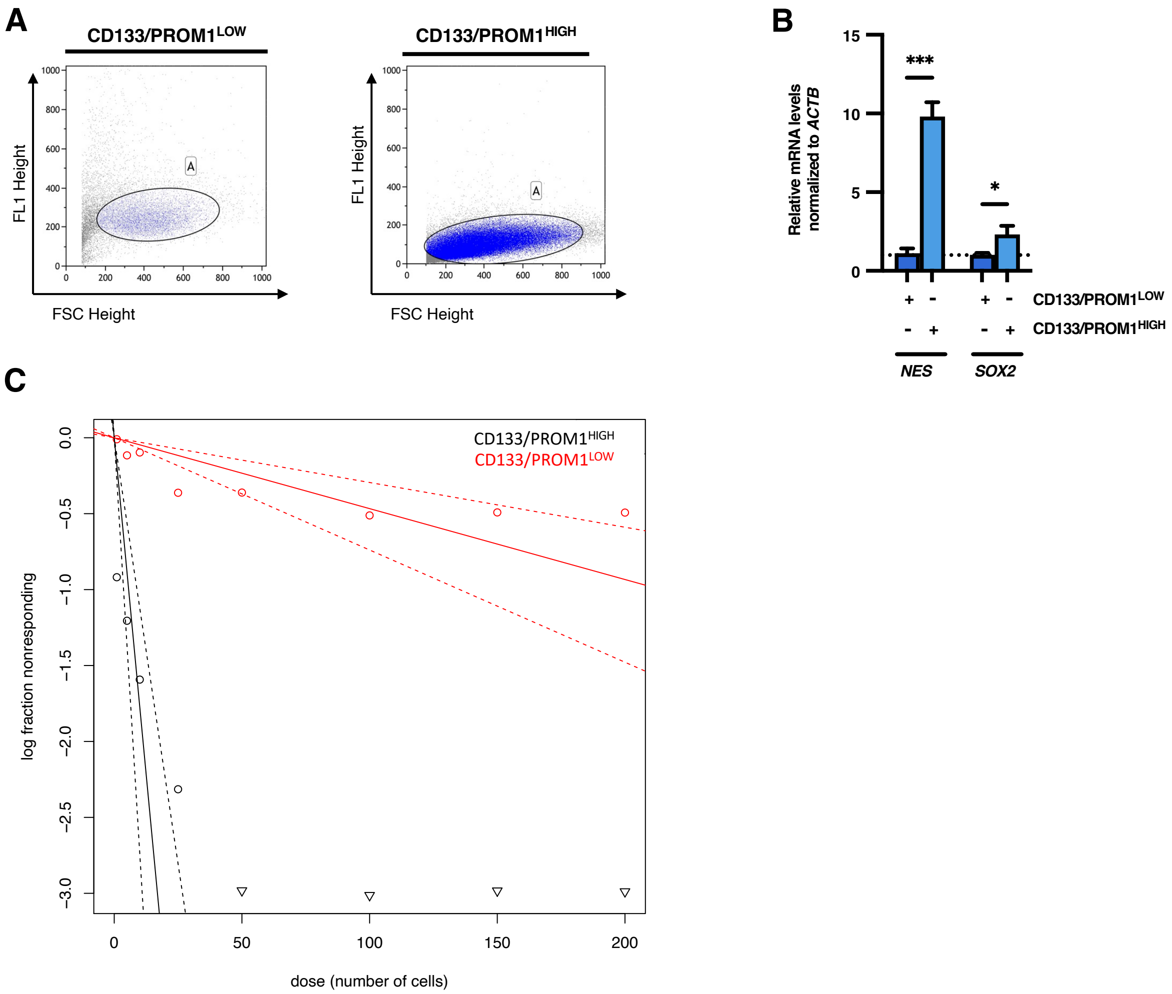

Supplementary Figure 1. (A) Gating strategy for flow cytometry analysis in Figure 1B. **(B)** CD133/PROM1^{LOW} and CD133/PROM1^{HIGH} patient-derived GBM cells were subjected to qRT-PCR analysis for the mRNA expression of *NES* and *SOX2*. Statistical analysis was performed using two-sided students t-test. *p < 0.05; **p < 0.01; ***p < 0.001; ns = non-significant. **(C)** Limiting Dilution Analysis plot generated using ELDA software from https://bioinf.wehi.edu.au/software/elda/.

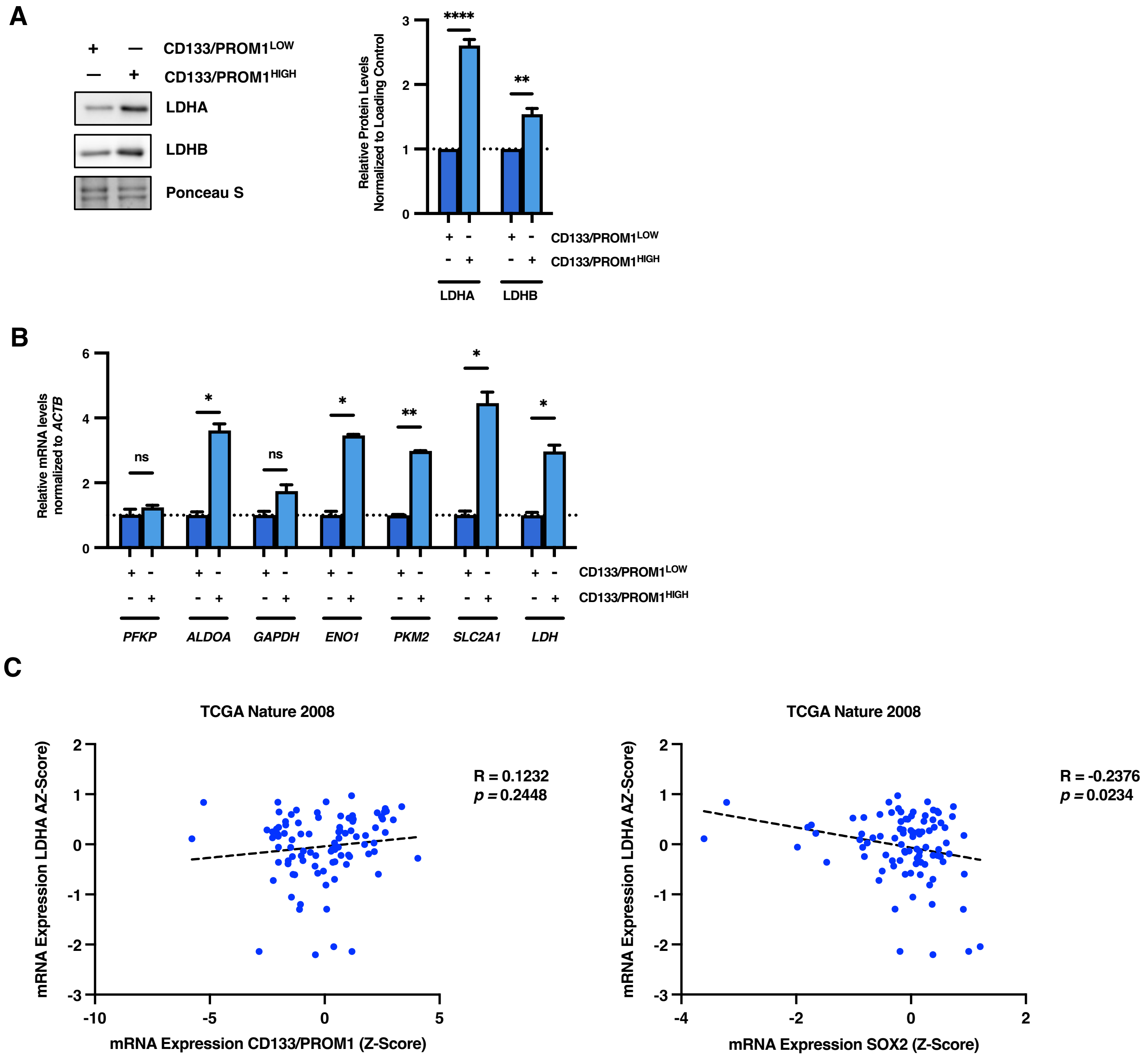

Supplementary Figure 2. (A) Western blot analysis comparing the expression of LDHA and LDHB in CD133/PROM1^{LOW} and CD133/PROM1^{HIGH} patient-derived GBM cells. Graph represents densitometry quantification of western blots from three independent experiments. Statistical analysis was performed using two-sided students t-test. *p < 0.05; **p < 0.01; ***p < 0.001; ns = non-significant. **(B)** CD133/PROM1^{LOW} and CD133/PROM1^{HIGH} patient-derived GBM cells were subjected to qRT-PCR analysis for the mRNA expression of *PFKP, ALDOA, GAPDH, ENO1, PKM2, SLC2A1,* and *LDH*. Statistical analysis was performed using two-sided students t-test. *p < 0.05; **p < 0.01; ***p < 0.001; ns = non-significant. **(C)** Bioinformatic analysis of mRNA expression from 91 GBM biospecimens from the TGCA pilot (Nature, 2008). Pearson correlation between the expression of the glycolytic enzyme *LDHA* and stemness markers (CD133/*PROM1* and *SOX2*) was performed and Pearson's r correlation coefficients were calculated. Statistical analysis was performed using two-sided student's t-test and exact p values are given.

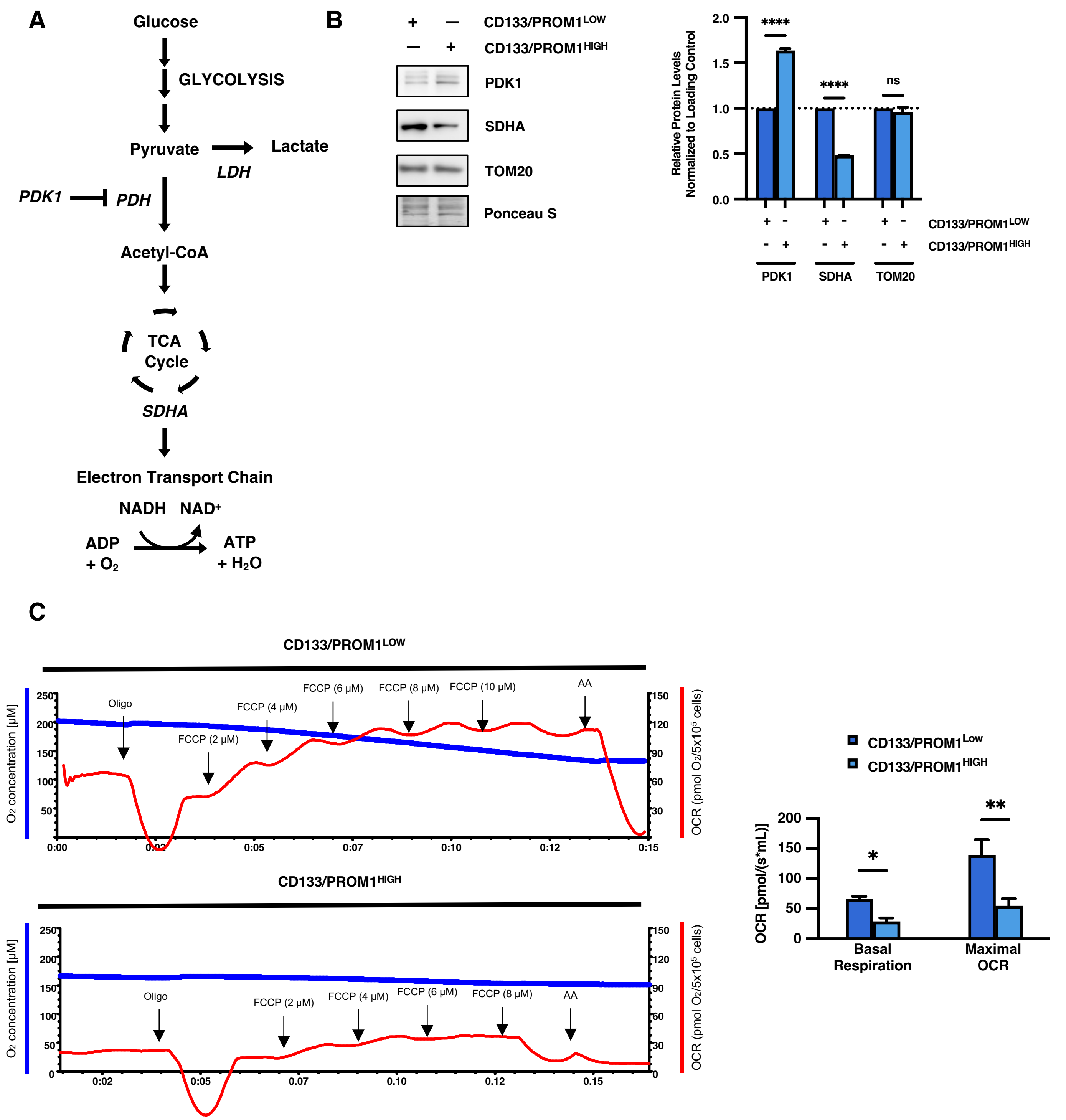

Supplementary Figure 3. (A) Schematic diagram depicting the enzymes which connect glycolysis with mitochondrial metabolism via the tricarboxylic acid cycle and the electron transport chain. **(B)** Western blot analysis comparing the expression of PDK1, SDHA, and TOM20 in CD133/PROM1^{LOW} and CD133/PROM1^{HIGH} patient-derived GBM cells. Graph represents densitometry quantification of western blots from three independent experiments. Statistical analysis was performed using two-sided students t-test. $*p < 0.05$; $**p < 0.01$; $***p < 0.001$; ns = non-significant. **(C)** CD133/PROM1^{LOW} and CD133/PROM1^{HIGH} patient-derived GBM cells were subjected to Oroboros respirometry with a representative tracing of Oroboros oxygraph. Graph represents of quantifications of basal respiration and maximal oxygen consumption rate (OCR). Statistical analysis was performed using two-sided students t-test. $*p < 0.05$; $**p < 0.01$; $***p < 0.001$; ns = non-significant

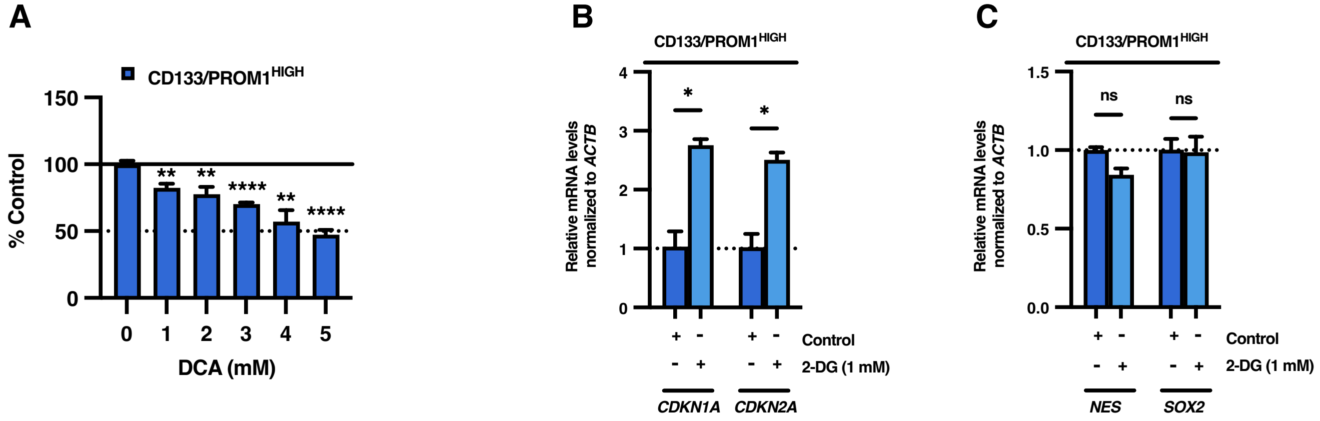

Supplementary Figure 4. (A) CD133/PROM1^{HIGH} patient-derived GBM cells were treated with increasing doses of dichloroacetate (DCA) from 1-5 mM. 24 hours post-treatment, cells were counted using trypan blue exclusion and then plotted as a percent of non-treated control cells. Statistical analysis was performed using two-sided students t-test. *p < 0.05; **p < 0.01; ***p < 0.001; ns = non-significant. **(B-C)** Non-treated controls and 2-DG (1mM) treated CD133/PROM1^{HIGH} patient-derived GBM cells were subjected to qRT-PCR analysis for the mRNA expression of **(B)** *CDKN1A* and *CDKN2A;* and (**C**) *NES* and *SOX2* . Statistical analysis was performed using two-sided students t-test. *p < 0.05; **p < 0.01; ***p < 0.001; ns = non-significant.

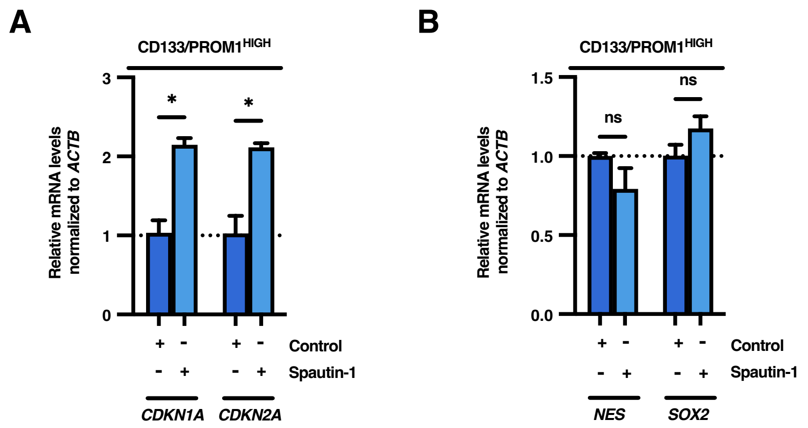

Supplementary Figure 5. (A-B) Non-treated controls and Spautin-1 (10 μM) treated CD133/PROM1^{HIGH} patient-derived GBM cells were subjected to qRT-PCR analysis for the mRNA expression of **(A)** *CDKN1A* and *CDKN2A;* and **(B)** *NES* and *SOX2*. Statistical analysis was performed using two-sided students t-test. *$p < 0.05$; **$p < 0.01$; ***$p < 0.001$; ns = non-significant.

SUPPLEMENTARY INFORMATION

Supplementary Table 1. List of commercial antibodies used in this study.

Target	Company	Catalogue Number
BMI1	Cell Signaling Technology	5856
SOX2	Cell Signaling Technology	3579
Nestin	Cell Signaling Technology	73349
PFKP	Cell Signaling Technology	8164
ALDOA	Cell Signaling Technology	8060
GAPDH	Santa Cruz Biotechnology	sc-47724
ENO1	Cell Signaling Technology	3810
PKM2	Cell Signaling Technology	4053
GLUT1/SLC2A1	Cell Signaling Technology	12939
LDH	Abcam	ab47010
LDHA	Cell Signaling Technology	3582
LDHB	Santa Cruz Biotechnology	sc-100775
PDK1	Cell Signaling Technology	3062
SDHA	Cell Signaling Technology	11998
TOM20	Cell Signaling Technology	42406
CDKN1A	Cell Signaling Technology	2947
CASP3	Cell Signaling Technology	9662
PARP	Santa Cruz Biotechnology	sc-8007
SQSTM1	Cell Signaling Technology	5114
MAP1/LC3A-I/II	Cell Signaling Technology	4599
MAP1/LC3B-I/II	Cell Signaling Technology	3868

Supplementary Table 2. List of human primer sequences used in this study.

Target	Forward 5'⟶ 3'	Reverse 5'⟶ 3'
NES	TCAAGATGTCCCTCAGCCTGGA	AAGCTGAGGGAAGTCTTGGAGC
SOX2	GGGAAATGGAGGGGTGCAAAAGAGG	TTGCGTGAGTGTGGATGGGATTGGTG
PFKP	AGGCAGTCATCGCCTTGCTAGA	ATCGCCTTCTGCACATCCTGAG
ALDOA	GTTATCAAATCCAAGGGCGGTGTT	AGTCAGCTCCGTCCTTCTTGTAC
ENO1	CTGGTGCCGTTGAGAAGGG	GGTTGTGGTAAACCTCTGCTC
PKM2	ATCGTCCTCACCAAGTCTGG	GAAGATGCCACGGTACAGGT
SLC2A1	TTGCAGGCTTCTCCAACTGGAC	CAGAACCAGGAGCACAGTGAAG
LDH	GCCCGACGTGCATTCCCGATTCCTT	GACGGCTTTCTCCCTCTTGCTGACG
CDKN1A	ACCTGGAGACTCTCAGGGTCG	TTAGGGCTTCCTCTTGGAGAAGAT
CDKN2A	CCCCTTGCCTGGAAAGATAC	AGCCCCTCCTCTTTCTTCCT
ACTB	TGACGTGGACATCCGCAAAG	CTGGAAGGTGGACAGCGAGG
